# Characterization and Value Assignment of a Monoclonal Antibody Reference Material, NMIJ RM 6208a, AIST-MAB

**DOI:** 10.3389/fmolb.2022.842041

**Published:** 2022-06-06

**Authors:** Tomoya Kinumi, Kazumi Saikusa, Megumi Kato, Reiko Kojima, Chieko Igarashi, Naohiro Noda, Shinya Honda

**Affiliations:** ^1^ National Metrology Institute of Japan (NMIJ), National Institute of Advanced Industrial Science and Technology (AIST), Tsukuba, Japan; ^2^ Manufacturing Technology Association of Biologics (MAB), Kobe, Japan; ^3^ Biomedical Research Institute, National Institute of Advanced Industrial Science and Technology (AIST), Tsukuba, Japan

**Keywords:** monoclonal antibody, biopharmaceutical, reference material, amino acid analysis, physicochemical property, antibody concentration

## Abstract

Monoclonal antibodies have been established as the largest product class of biopharmaceuticals. Since extensive characterization is required for development and quality control of monoclonal antibody, a widely available reference material (RM) is needed. Herein, a humanized IgG1κ monoclonal antibody reference material, RM 6208-a, AIST-MAB, was established by the National Metrology Institute of Japan, National Institute of Advanced Industrial Science and Technology (NMIJ/AIST). The monoclonal antibody solution was produced as a pharmaceutical grade using a Chinese hamster ovary-derived cell line. The assigned indicative value represents the concentration of the antibody with a heterotetrameric structure including oligomeric forms, determined by an amino acid analysis using isotope dilution mass spectrometry, and their homogeneity and stability were assessed. In addition to antibody concentration, various physicochemical properties, including peptide mapping data, charge variants, and aggregates, were examined. This RM is intended for use in validation of analytical procedures and instruments such as a system suitability test for quantification of antibody. It is also intended for comparing and evaluating the results of antibody analyses across analytical methods and analytical laboratories such as inter-laboratory comparison. Both the material and the set of data from our study provide a tool for an accurate and reliable characterization of product quality attributes of monoclonal antibodies in biopharmaceutical and metrology communities.

## 1 Introduction

Monoclonal antibodies have dominated the biopharmaceutical market among various modalities. The number of approved antibody drugs in the US and the EU has increased nearly three-fold from 2010 to 2019 (Kaplon et al., 2020). In 2020, it is reported that 15 antibody therapeutics have been approved worldwide (Kaplon and Reichert, 2021). Because the production of antibody drug utilizes the biosynthetic process of living organisms, the design and management of the development and manufacturing process affect the quality of the final product directly. Moreover, the quality among different production lots differs considerably even if the same production cells are used, and properties of the follow-on biologics (biosimilars) made by different manufacturers differ from those of the original products.

Therefore, physicochemical properties such as structural heterogeneity and aggregation should be evaluated in detail to demonstrate product consistency and equivalence. To address this situation, the International Council for Harmonisation of Technical Requirements for Pharmaceuticals for Human Use (ICH) Q6B provides guidelines for specification on the characterization of biopharmaceuticals, and the establishment of acceptance criteria as well as analytical procedures (ICH Q6B, 1999). In terms of primary structure, structural heterogeneity includes posttranslational modifications (PTMs), such as glycosylation, disulfide bond mismatch, deamidation of asparagine residues, oxidation of methionine and tryptophan, glycosylation, and cleavage of the polypeptide chain (Liu et al., 2008; Beck et al., 2013). The variety of higher-order structures, such as denaturation, misfolding, and aggregation, should also be evaluated. The results of these quality attributes may vary depending on the measurement method, and many technologies are under development (Le Basle et al., 2020).

National metrology institutes (NMIs) have been leading to establish traceable measurement to a known reference, particularly focusing on the development of a reference material (RM) traceable to Système International d'Unités (SI). The provision of a reliable RM and calibration service by NMIs is defined by international standards, such as ISO 17025 and ISO 17034, which provide requirements to support best practices in production and maintenance of the RM and quality system (ISO 17034, 2016; ISO/IEC 17025, 2017). Although different platforms exist among biopharmaceutical and metrology communities, there is need for a well-characterized and widely available monoclonal antibody RM that validates methods and measurement results for the development of an analytical technology. Among the various properties, antibody concentration is the fundamental basis for many properties, including physicochemical properties, biological activities, and immunochemical properties, as well as any quantitative assays of protein–protein interaction and protein–ligand interaction parameters such as binding constant and enzyme activity.

The National Institute of Standards and Technology (NIST), first released an antibody RM, namely, NISTmAb (RM 8671), which is a recombinant humanized IgG1κ solution, and whose assigned antibody concentration was determined by absorption spectrometry (Schiel et al., 2018) as the reference value, and size heterogeneity (Turner et al., 2018) and charge heterogeneity (Turner and Schiel, 2018) were also assigned as its reference values. Moreover, this material provides a case study of important quality characteristics measured through collaborative measurements involving pharmaceutical companies and research institutes, in addition to the reference values determined independently by NIST (Schiel et al., 2014; 2015a; 2015b).

In antibody analysis, there is an increasing demand for a widely available and metrologically reliable monoclonal antibody RM. The National Metrology Institute of Japan/National Institute of Advanced Industrial Science and Technology (NMIJ/AIST) has developed a RM of a monoclonal antibody solution, namely, NMIJ RM 6208a, AIST-MAB. This RM is a recombinant monoclonal antibody (humanized IgG1κ) solution in 10 mmol/L potassium phosphate buffer (pH 7.0) produced from Chinese hamster ovary (CHO)–derived cell line. The assigned indicative value of this material represents the antibody concentration with a heterotetrameric structure including oligomeric forms determined by an amino acid analysis. We also characterized a wide variety of physicochemical properties of this material in addition to the antibody concentration. This RM is intended for use in validation of analytical procedures and instruments such as a system suitability test for quantification of antibody. It is also intended for comparing and evaluating the results of antibody analyses across analytical methods and analytical laboratories such as inter-laboratory comparison. Thus, the material can be used for various quality analyses rather than for a specific biopharmaceutical product.

Herein, we report the development of AIST-MAB. The assigned indicative value represents the concentration of the antibody, which was determined by two independent amino acid analyses based on isotope dilution mass spectrometry using liquid phase and gas phase hydrolyses with liquid chromatography–tandem mass spectrometry (LC/MS/MS). The amino acid analysis using isotope dilution mass spectrometry has been used as a “gold standard” for the method of traceable protein quantification (Burkitt et al., 2008; Munoz et al., 2011) and for the value assignment of various certified reference materials from NMIJ, including C-peptide (Kinumi et al., 2012), C-reactive protein (Kato et al., 2015), and human serum albumin (Kinumi et al., 2017). Along with a quantitative analysis by the amino acid analysis, homogeneity and stability tests were conducted, and values were assigned in accordance with ISO 17034. The resulting indicative value of this material has been determined to be 5.00 (± 0.19) g/L, the number following ± represents the expanded uncertainty with a coverage factor *k* = 2 giving a level of confidence of approximately 95%. Moreover, we describe the value assignment as well as the analytical results for various physicochemical properties of AIST-MAB in detail.

## 2 Materials and Methods

### 2.1 Materials

Acetonitrile and formic acid for an LC-MS analysis (LC-MS grade) were obtained from Fujifilm Wako Pure Chemical (Japan). The reagents used were of the highest grade obtained from Fujifilm Wako Pure Chemical (Japan), unless otherwise stated. All the aqueous solutions and solvents were prepared using ultrapure water purified with the Milli-Q purification system (Merck Millipore, USA).

### 2.2 Preparation of the Candidate Material

The candidate material was expressed using the CHO-derived cell line. After fermentation of the antibody-producing CHO cell line in a serum-free culture medium for 7 days using the Allegro XRS 25 Bioreactor (Pall, USA), the culture supernatant was prepared using the Millistak Pod depth filtration system (EMD Millipore, USA) to remove cells and debris. Thereafter, it was purified further by three-step chromatographic technique using protein A affinity (RTP MabSelect SuRe, Cytiva, USA), anion-exchange (RTP Capto Q, Cytiva, USA), and cation-exchange (RTP Capto S, Cytiva, USA) columns. The purified material was treated using the Planova 20 N virus removal filter (Asahi Kasei Medical, Japan), concentrated through Pellicon ultrafiltration (EMD Millipore, USA) and buffer exchange to 10 mmol/L potassium phosphate buffer (pH 7.0) in a good manufacturing practice (GMP) grade facility. The raw material (1 ml) was dispensed into polypropylene vials sterilely using a Microlab STARlet 8ch liquid handling system (Hamilton, USA) with a FluidX XSD-48Pro automated capper/decapper system (Azenta Life Sciences, USA) and stored at −80°C. These processes were performed at Manufacturing Technology Association of Biologics at Kobe, Japan.

### 2.3 Structural Analyses *via* Mass Spectrometry

LC-MS for structural analyses was performed using a maXis II electrospray ionization quadrupole time-of-flight mass spectrometer (Bruker, Germany) in a positive ion mode coupled with an LC-30A Nexera HPLC system (Shimadzu, Japan). The data were analyzed using Data Analysis 4.3 software (Bruker, Germany).

#### 2.3.1 Intact Mass Spectrometry

We injected 1 μl of the candidate RM into an LC-MS system using the AQUITY UPLC Protein BEH C4 column (1.7 μm, 2.1 mm diameter × 100 mm length, Waters, USA), mobile phase A: 0.1% formic acid/H_2_O, B: 0.1% formic acid/acetonitrile, flow rate: 0.2 ml/min, column temperature: 60°C, and gradient condition: 5–15 %B in 15 min. The mass spectrometer was operated under the following conditions: capillary voltage: 4500 V, nebulizer gas: 1.2 bar, dry gas: 6 L/min, isCID: 30 eV, quadrupole ion energy 4 eV, collision energy: 8 eV, mass range: *m/z* 500–3000, and spectra rate: 3 Hz. The mass spectrometer was calibrated using ESI-L Low concentration tuning mix (G1969-85000, Agilent Technologies, USA).

#### 2.3.2 LC-MS Measurement for Light and Heavy Chains

To 5 μl of the candidate RM, 50 μl of water and 5 μl of 500 mmol/L TCEP [tris(2-carboxyethyl)phosphine] were added and incubated at 37°C for 2 h. A volume of 2 μl of material was injected into the LC-MS system. The gradient condition used was 5 %B 2 min, 5–15 %B 1 min, 15–25 %B 3 min, and 25–35 %B 15 min, and other measurement conditions were same as intact mass spectrometry.

#### 2.3.3 LC-MS Measurement for IdeS Digestion

To 50 μl of the candidate RM, 50 μl of 50 mmol/L phosphate buffer and 4 μl of IdeS protease (IdeS FabRICATOR, 270 U, Sigma-Aldrich, USA) were added and incubated at 37°C for 1 h. The reduced form was obtained by adding an additional 5 μl of 500 mmol/L TCEP prior to IdeS digestion and incubation at 37°C for 1 h. A volume of 3 μl of the resulted solution was injected into the LC-MS system. The measurement conditions were same as those used for light and heavy chains.

#### 2.3.4 Peptide Mapping

To 100 μl of 8 mol/L guanidine hydrochloride, 1 mmol/L ethylenediaminetetraacetic acid, 250 mmol/L Tris-HCl (pH 8.0), and 20 μl of the candidate RM were added, and then 5 μl of 500 mmol/L dithiothreitol (DTT, Sigma-Aldrich, USA) was added and incubated at 37°C for 1 h. Thereafter, the reaction mixture was incubated for 1 h in the dark at room temperature with 12 μl of 500 mmol/L iodoacetic acid, followed by 5 μl of 500 mmol/L DTT. After desalting using a NAP-5 gel filtration column (Cytiva, USA), trypsin or Lys-C (lysylendopeptidase) or Glu-C (mass spectrometry grade, FUJIFILM Wako Pure Chemical, Japan) was added to a 300 μl fraction of desalted NAP-5 elutant at 1:25 (enzyme: substrate) ratio of protein content and incubated at 37°C overnight for trypsin, Lys-C, and Glu-C, or 1 h for trypsin digestion. The reaction was terminated by adding 1 μl trifluoroacetic acid, and then 5 μl was injected into LC-MS/MS for the measurement. For peptide mapping by trypsin in a non-reduced condition, the sample was prepared using the aforementioned procedure without reduction and alkylation steps by DTT and iodoacetic acid. The measurement conditions were as follows: the chromatography column used was AQUITY UPLC Peptide BEH C18 (3.5 μm, 2.1 mm diameter × 150 mm length, Waters, USA); mobile phase A: 0.1% formic acid/H_2_O; B: 0.1% formic acid/acetonitrile; flow rate: 0.2 ml/min; column temperature: 45°C; and gradient condition: 2 %B 3 min, 2–7 %B 1 min, 7–10 %B 4 min, 10–25 %B 32 min, and 25–38 %B 15 min. The mass spectrometer was operated under the following conditions: capillary voltage: 4500 V, nebulizer gas: 1.2 bar, dry gas: 6 L/min, isCID: 0 eV, quadrupole ion energy 5 eV, collision energy: 10 eV, mass range: *m/z* 100–3500, and spectra rate: 5 Hz. The LC-MS/MS data were acquired by a data-dependent MS/MS mode; precursor ions: number of precursor 2, active exclusion exclude after two spectra, release after 0.1 min, and reconsider precursor 2.0. The mass spectrometer was calibrated using 5 mmol/L ammonium formate in 50% isopropanol/water by internal calibration.

### 2.4 Chromatography

Cation exchange chromatography (CEX) was performed using an LC-20A Prominence HPLC system with an ultraviolet (UV) detector (Shimadzu, Japan) and a BioPro IEX SF column (5 μm, 4.6 mm diameter × 100 mm length, YMC, Japan). A volume of 5 μl of the candidate RM was injected into the LC-UV system. The measurement conditions were as follows: mobile phase A: 20 mmol/L 2-(*N*-morpholino) ethanesulfonic acid (MES) (pH 6.0), mobile phase B: 20 mmol/L MES, 200 mM NaCl (pH 6.0), flow rate: 0.5 ml/min, column temperature: 30°C, and gradient condition: 10–80 %B 30 min. Absorbance was detected at a wavelength of 215 nm. The performance criteria were set to 5.6 (± 1.2) min for α-chymotrypsinogen A from bovine pancreas (Sigma-Aldrich, USA), and 23.2 (± 0.9) min for equine myoglobin (Serva, USA) based on two sigmas of averaged retention time.

Size-exclusion chromatography (SEC) was performed using the LC-UV system used for CEX. The chromatography column used was a TSK gel G3000SW_XL_ (5 μm, 7.8 mm diameter × 300 mm length, TOSOH, Japan). A volume of 10 μl of the candidate RM was injected into the LC-UV system. The measurement conditions were as follows: 100 mmol/L sodium phosphate buffer containing 100 mmol/L Na_2_SO_4_ (pH6.8) in isocratic elution, flow rate: 0.4 ml/min, and column temperature: 25°C. Absorbance was detected at a wavelength of 280 nm. The system performance was evaluated using molecular weight marker proteins for SEC from Oriental Yeast (Japan) consisting of five proteins. The performance criteria based on two sigmas of averaged retention time were set to 20.0 (± 0.1) min for glutamate dehydrogenase, 23.1 (± 0.1) min for lactose dehydrogenase, 24.9 (± 0.1) min for enolase, 27.2 (± 0.1) min for myokinase, and 28.9 (± 0.1) min for cytochrome C.

### 2.5 Electrophoreses

Microchip electrophoresis was performed using the LabChip GXII Touch24 electrophoresis system (PerkinElmer, USA). The sample solution was prepared by using 2 μl of diluted the candidate RM at approximately 2.5 mg/g using the Protein Express Assay Reagent Kit (PerkinElmer, USA) at 70°C for 10 min, according to the manufacturer’s instruction. The results of the three lanes were averaged to obtain the measurement results. Measured molecular masses were calibrated using a molecular weight marker supplied with the Protein Express Kit (PerkinElmer, USA). The standard deviation of the measured molecular masses was confirmed to be less than 5%.

Capillary isoelectric focusing was performed using an iCE3/Alcott720NV capillary isoelectric focusing system with Fc cartridge (100 μm diameter × 50 mm length, ProteinSimple, USA). The sample solution was prepared with 0.4 mg/mL as the final concentration of the candidate RM in 4% pharmalyte (pH 3–10), 0.35% methylcellulose, and 10 mmol/L arginine solution. Electrophoresis conditions were 1500 V for 1 min while prefocusing and 3 kV for 4.5 min when focusing on detection at 280 nm. The measurements were repeated thrice. Samples were measured after confirming that the isoelectric points of human hemoglobin (Sigma-Aldrich, USA) were within 7.1 ± 0.1 and 7.2 ± 0.1, and the variation in triplicate measurements of the peak height of a high pI marker (pI 9.77) supplied with High pI marker (102219, ProteinSimple, USA) was less than 10%.

### 2.6 Glycan Mapping

Sample preparation including hydrolysis by peptide *N*-glycosidase F (PNGaseF) and 2-aminobezamide (2-AB) derivatization of glycans from the candidate RM was performed using the EZGlyco mAb-N Kit with 2-AB (BS-X4410, Sumitomo Bakelite, Japan). A volume of 2 μl of the resulted glycan mixture solution obtained with 8 μl of candidate RM was injected into an LC-fluorescence detection (LC-FL) system for analysis. The LC-FL system used was a Nexera 30A HPLC system with a fluorescence detector (Shimadzu, Japan) with an AQUITY UPLC Protein BEH Amide column (1.7 μm, 2.1 mm diameter × 150 mm length, Waters, USA). The measurement conditions were as follows: mobile phase A: 100 mmol/L ammonium formate (pH 4.5), B: acetonitrile, flow rate: 0.2 ml/min, column temperature: 45°C, gradient condition: 75–50 %B 50 min, fluorescent detection: excitation at 330 nm, and detection at 420 nm.

### 2.7 Analysis of Impurities

#### 2.7.1 Host Cell–Derived Protein Assay

Residual amount of HCP was quantified by enzyme-linked immunosorbent assay (ELISA) using a commercial ELISA kit, CHO Host Cell Proteins 3rd Generation F550 (Cygnus Technologies, USA), according to the manufacturer’s instruction. In brief, 100 μl of Anti-CHO-HRP was added into each well of an anti-CHO–coated 96-well plate followed by adding 50 μL of triplicate samples (n = 3), standards, or blank controls were added in each well and incubated for 3 h at room temperature on a plate shaker. Thereafter, the plates were washed four times with 350 μl of wash solution. To each well, 100 μl of HRP substrate, 3,3′,5,5′-tetramethyl-benzidene, was added and incubated at 25°C for 30 min before adding the stop solution. The absorbance of the reactant at 450 nm in each well was recorded using an Enspire 2300 multilabel plate reader (PerkinElmer, USA).

#### 2.7.2 Protein A Assay

Residual amount of protein A was quantified *via* the amplified luminescent proximity homogeneous assay (AlphaLISA®) using a commercial kit, Residual Protein A kit AL287 (PerkinElmer, USA), according to the manufacturer’s instruction (Protocol 2). In brief, 60 μl of triplicate samples (n = 3) or standards were mixed with 120 μl of 3× dissociation buffer in a tube, heated at 98°C for 60 min, and centrifuged for 5 min at ≥ 200 *g*. Thereafter, 10 μl of supernatants were transferred to each well of a 96-well plate, and then 20 μl of 5× anti-protein A acceptor beads were added and the mixture was incubated at room temperature for 30 min. Subsequently, 20 μl of 5× biotinylated antibody anti-protein A were added into each well, followed by incubation at room temperature for 60 min, after which 50 μl of 2× SA-donor beads were added, and the mixture was incubated at room temperature for 30 min in the dark. The emission from the donor beads at 615 nm was recorded using an Enspire 2300 multilabel plate reader.

#### 2.7.3 Host Cell–Derived DNA Assay

The residual amount of host cell–derived DNA was quantified *via* the quantitative polymerase chain reaction (qPCR) using a StepOnePlus Real-Time PCR System (Applied Biosystems, USA). First, DNA was extracted using a commercial kit, DNA Extraction and Amplification Kit D555T (Cygnus Technologies, USA), according to the manufacturer’s instruction. In brief, 500 μl of duplicate samples (n = 2), samples for addition recovery tests (n = 2), standard, and control were placed in a 2 ml microfuge tubes, and then 25 μl of 1× Proteinase K was added to them. The tubes were gently vortexed for 5 s, incubated at 60°C for 30 min, and centrifuged for 1 min at 10,000 rpm. After this, 500 μl of extraction buffer was added to the tubes, which were vortexed for 10 s, and 1 ml of precipitation buffer was added to the tubes and vortexed for 10 s. The tubes were incubated for 10 min and centrifuged at 10,000 rpm for 10 min. After decanting supernatants, 1.5 ml of DNA wash buffer was introduced to each tube, followed by vortexing for 5 s, incubating at room temperature for 20 min, and centrifuging at 10,000 rpm for 5 min. After decanting supernatants again, pellets were re-suspended in 50 μl of DNA TE buffer and incubated at 50°C for 2–3 min to dissolve CHO DNA completely. Thereafter, DNA amplification was performed using TB Green Premix Ex Taq GC (TaKaRa, Japan) and custom primers for glyceraldehyde-3-phosphate dehydrogenase (GAPDH) gene as the target region. In brief, 10 μl of samples, samples for addition recovery tests, standard, and control were added to 25 μl of the PCR mixtures containing SYBR Premix Ex Taq GC, Rox reference dye, and GAPDH primers and transferred to each well of a qPCR assay plate. The PCR amplification parameters were as follows: heating stage: 95°C for 30 s, cycling stage: 45 cycles at 95°C for 10 s and 60°C for 30 s, and melting curve stage: 95°C for 15 s followed by 60°C for 60 s and 95°C for 15 s. The samples were tested in quadruplicate (n = 4) in the qPCR measurement.

#### 2.7.4 Endotoxin Assay

Endotoxin was quantified by turbidimetry using Toxinometer ET-6000 (FUJIFILM Wako Pure Chemical, Japan) with Limulus ES-II Single Test (FUJIFILM Wako Pure Chemical, Japan), according to Japanese Pharmacopoeia (Japanese Pharmacopoeia 2016). In brief, 200 μl of duplicate samples (n = 2) or standards were mixed with the Limulus amebocyte lysate agents, incubated at 37 ± 1 °C for 60 ± 2 min in the toxinometer, and subsequently subjected to turbidimetric analysis.

### 2.8 Preparation of Standard Solutions and Blend Mixtures for Amino Acid Analyses

The standard solutions of natural amino acids were gravimetrically prepared by dissolving the following NMIJ CRMs in 10 mmol/L HCl: l-aspartic acid (NMIJ CRM 6027a), l-glutamic acid (NMIJ CRM 6026a), l-proline (NMIJ CRM 6016a), l-valine (NMIJ CRM 6015a), l-isoleucine (NMIJ CRM 6013a), l-leucine (NMIJ CRM 6012a), l-phenylalanine (NMIJ CRM 6014a), and l-alanine (NMIJ CRM 6011a). The standard mixture of amino acids was gravimetrically prepared by mixing the standard solution of each amino acid in the same molar ratio of as that of each amino acid composition in the monoclonal antibody molecule. The following isotopically labeled amino acids (Cambridge Isotope Laboratories, USA) were used as the internal standard: l-^13^C_4_^15^N-Asp, l-^13^C_5_^15^N-Glu, l-^13^C_5_^15^N-Pro, l-^13^C_6_^15^N_2_-Lys, l-^13^C_5_^15^N-Val, l-^13^C_6_^15^N-Ile, l-^13^C_6_^15^N-Leu, l-^13^C_9_^15^N-Phe, and l-^13^C_3_^15^N-Ala. The candidate RM (0.1 ml) and the mixture of isotopically labeled amino acids (0.1 ml) were gravimetrically dispensed into a glass vial. The dispensed solutions were dried with gentle nitrogen flow. Calibration blends were gravimetrically prepared by mixing the standard mixture of natural amino acids and mixture of isotopically labeled amino acid solutions. The calibration blends (0.2 ml) were dispensed into a glass vial and dried by gentle nitrogen flow.

### 2.9 Amino Acid Analyses

For liquid-phase hydrolysis, dried sample blend and calibration blend in a glass vial were dissolved by adding 0.2 ml of 6 mol/L HCl and 0.1% phenol. Samples were hydrolyzed at 150°C for 1 and 3 h, 160°C for 1 and 3 h, and 170°C for 1 and 3 h using an ETHOS One microwave digestion system (Milestone SRL, Italy) after purging with nitrogen. The hydrolysate was analyzed by pre-column derivatization with *N*-butylnicotinic acid *N*-hydroxysuccinimide ester iodide and measured *via* LC-MS/MS under the selected ion monitoring (SRM) mode.

For gas-phase hydrolysis, the dried sample in the glass vial was hydrolyzed under the gas phase by 6 mol/L HCl and 2% phenol at 130°C for 18, 24, and 48 h as well as 150°C for 18, 24, and 48 h using a Pico-tag workstation hydrolysis system (Waters, USA) after purging with nitrogen. The hydrolysate was analyzed by hydrophilic interaction chromatography (HILIC)-MS/MS under the SRM mode. The detailed measurement conditions for amino acid analyses are described in the supplementary material.

### 2.10 Homogeneity and Stability Tests

Homogeneity was assessed by measuring the relative area percentage of the main peak obtained by CEX in triplicate for twelve selected vials.

Stability was evaluated as acceleration (stored at 4, or 25°C), long-term (stored at −80°C), short-term (stored at −20°C), and freeze-thaw cycles (up to five times) tests. The long-term stability test was performed *via* CEX and UV absorption at 280 nm (see the below section) using a previous lot of the preceding product. The acceleration, short-term, and freeze-thaw cycles tests were performed *via* CEX, SEC, and UV absorption at 280 nm using the candidate RM.

### 2.11 Density Measurement

The density of the candidate RM was measured using a vibration type of the density meter (DMA5000EX, Anton Paar, Austria) in triplicate. Calibration was performed using dried air and pure water density standard (QAT182462, Kyoto Electronics, Japan).

### 2.12 Ultraviolet (UV) Absorbance Measurement

UV absorbance measurement was performed using a UV-2550 spectrophotometer (Shimadzu, Japan) calibrated with potassium dichromate solution and optical filter, according to the Japanese Industrial Standards (JIS) K0115 (JIS K0115, 2004). A quartz cell with nominal 1-mm optical path length (1/Q/1, Starna Scientific, England) was used to measure the absorbance of the candidate RM at 280 nm and at 1 nm of the band path. Absorbance was measured using a double-beam spectrophotometer without a reference cell, using the same procedure as that used for a single-beam type instrument. A solvent blank sample was measured first, then the sample solution was measured using the same optical cell, and their difference was used to determine the absorption of the sample solution.

## 3 Results and Discussion

### 3.1 Assessment of Size Heterogeneity and Charge Variants

**FIGURE 1:**
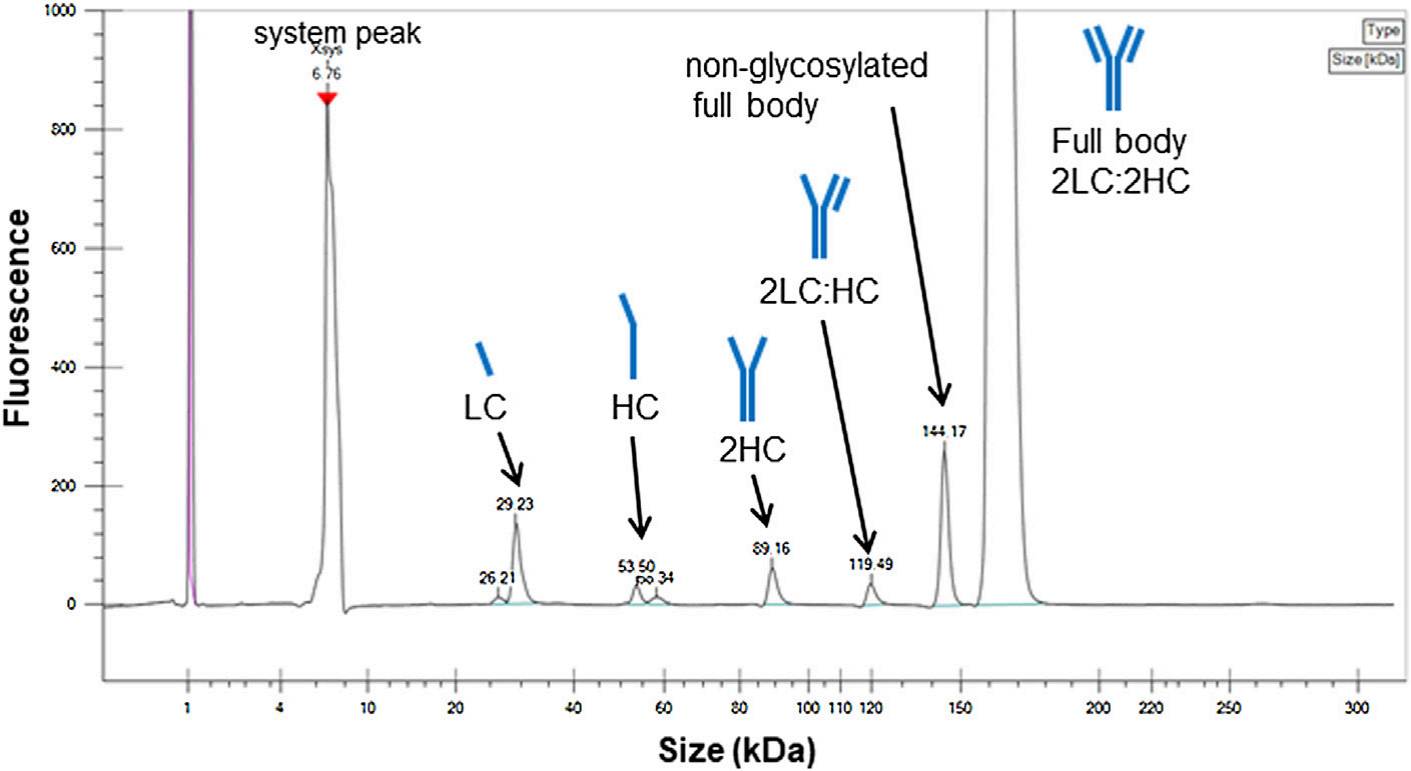
Electropherogram of the candidate reference material (RM) analyzed by sodium dodecyl sulfate microchip electrophoresis under denatured and non-reducing condition. Peak assignments are based on the migration times. LC, light chain; HC, heavy chain.

The desired target structure of IgG1 is a heterotetrameric structure with two heavy chains (HC) and two light chains (LC; full body, 2LC:2HC). However, biopharmaceutical products usually contain free subunit chains, fragments of each subunit, or products with insufficient tetramer formation owing to disulfide bond scrambling as the size variants (Gaza-Bulseco and Liu, 2008; Leblanc et al., 2017; Turner and Schiel, 2018). These small size variants were analyzed *via* electrophoresis under denatured and non-reducing condition using a microchip electrophoresis system, whose result is presented in Figure 1.

**FIGURE 2:**
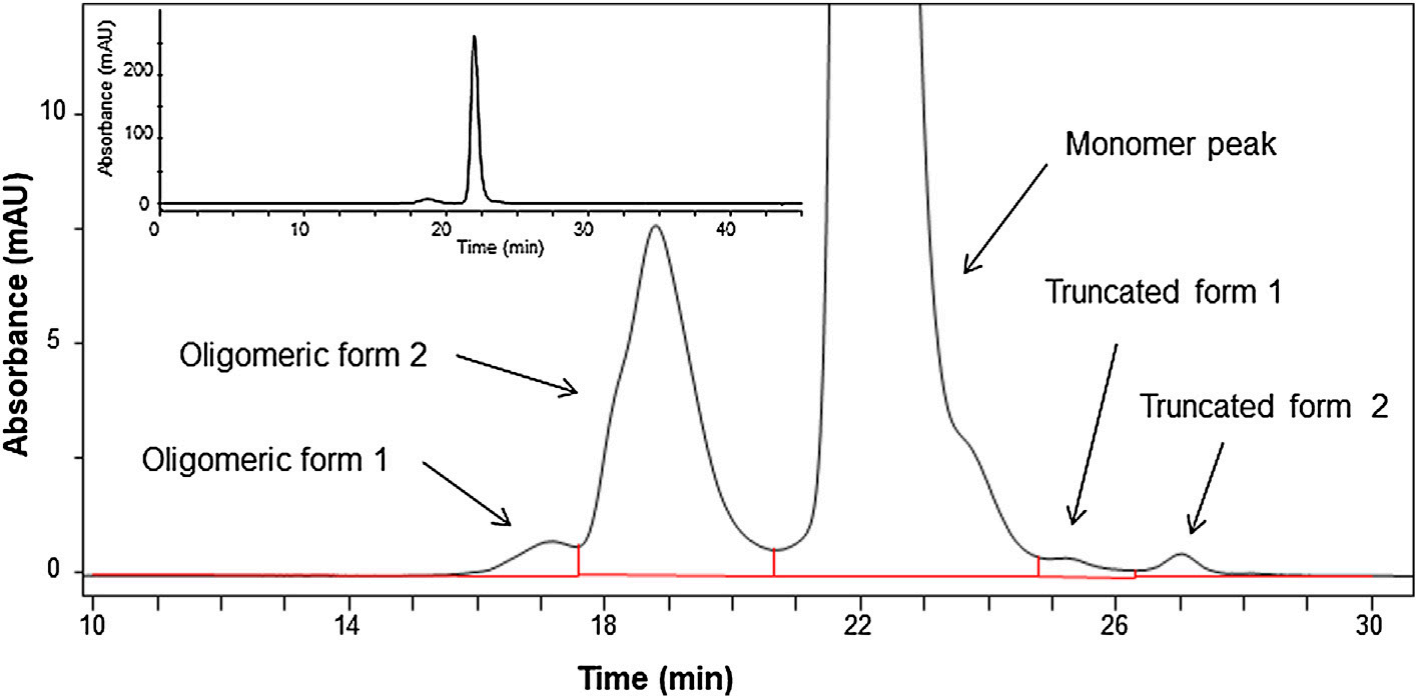
Chromatogram of the candidate reference material (RM) analyzed by size-exclusion chromatography (SEC) monitored at 280 nm. Insert shows expanded chromatogram.

Peaks were assigned according to the molecular size, indicating free LC, HC, partial molecules, 2HC and 2LC:HC, and non-glycosylated. The peak area ratio of the full body was estimated to be 93%, which was considered to be a minimum value. This is because the sample may dissociate into the partial molecules depending on the sample preparation conditions, and the area ratio may vary depending on the measurement conditions. In addition to the evaluation *via* electrophoresis, the size heterogeneity was assessed *via* SEC, which enabled to analyze fragmented products to aggregated macro molecule. As shown in Figure 2, the chromatogram obtained *via* SEC with UV detection exhibited four peaks assigned as oligomers (oligomeric form 1 and 2) and truncated products (truncated form 1 and 2) with the area percentage of (93.7 ± 0.2) % of the monomer peak. The entities regarding oligomeric forms 1 and 2 were attributed to trimer and dimer with area percentage of (0.42 ± 0.01) % and (5.72 ± 0.14) %, respectively. The entities of truncated forms 1 and 2 were (0.16 ± 0.01) %, which was negligibly small compared to the sum of the monomeric and oligomeric forms.

**FIGURE 3:**
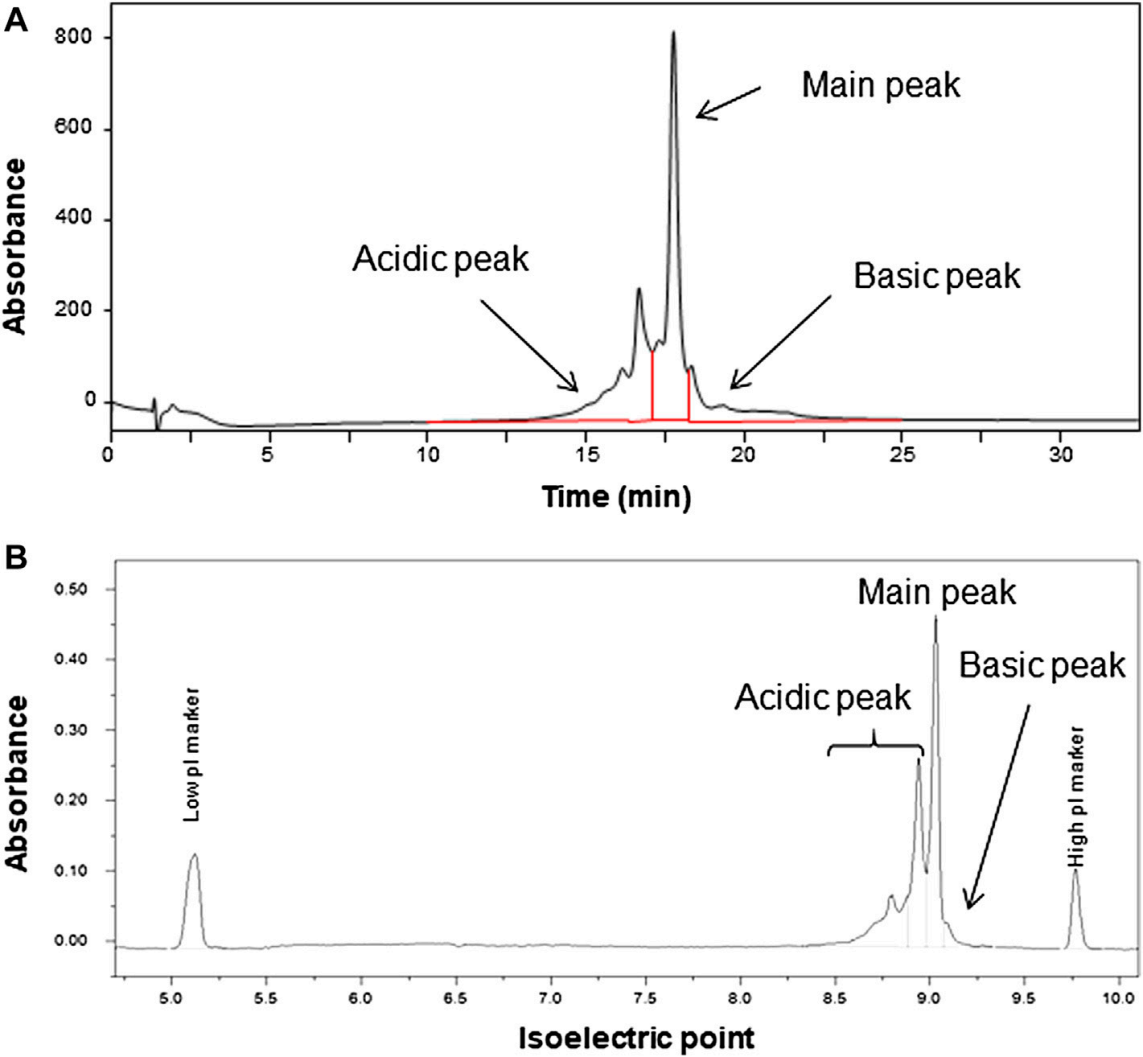
Chromatogram analyzed by cation exchange chromatography (CEX) **(A)** and electropherogram analyzed by capillary isoelectric focusing **(B)** of the candidate reference material (RM).

The heterogeneity owing to charge variants was assessed *via* CEX and capillary isoelectric focusing (cIEF). The net charge of the monoclonal antibody may depend on the PTMs including deamidation and conformational changes, resulting in the charge distribution of the molecule (Du et al., 2012; Fekete et al., 2015; Turner and Schiel, 2018). The chromatogram obtained *via* CEX is shown in Figure 3A. The chromatographic peaks on the chromatogram were divided into three, and the area percentages of each peak were (35.9 ± 0.1) % for the acidic peak (47.8 ± 0.2) % for the main peak, and (16.4 ± 0.1) % for the basic peaks. The electropherogram obtained *via* cIEF is shown in Figure 3B, and exhibited area percentages of indicated (54.3 ± 0.9) % for the acidic peak (42.1 ± 0.6) % for the main peak, and (3.6 ± 0.3) % for basic peak. The charge distribution was evaluated using two independent methods with different measurement principles, and results indicated similar main peak contents with area percentages of 47.8 and 42.1%, although not identical. The isoelectric point of the candidate RM obtained *via* cIEF was 9.03.

### 3.2 Structural Analyses

Confirmation of the primary structure and identification of the PTM were performed by mass spectrometry–based analyses, including intact mass spectrometry, middle-down structural characterization, and peptide mapping.

#### 3.2.1 Intact Mass Spectrometry

**FIGURE 4:**
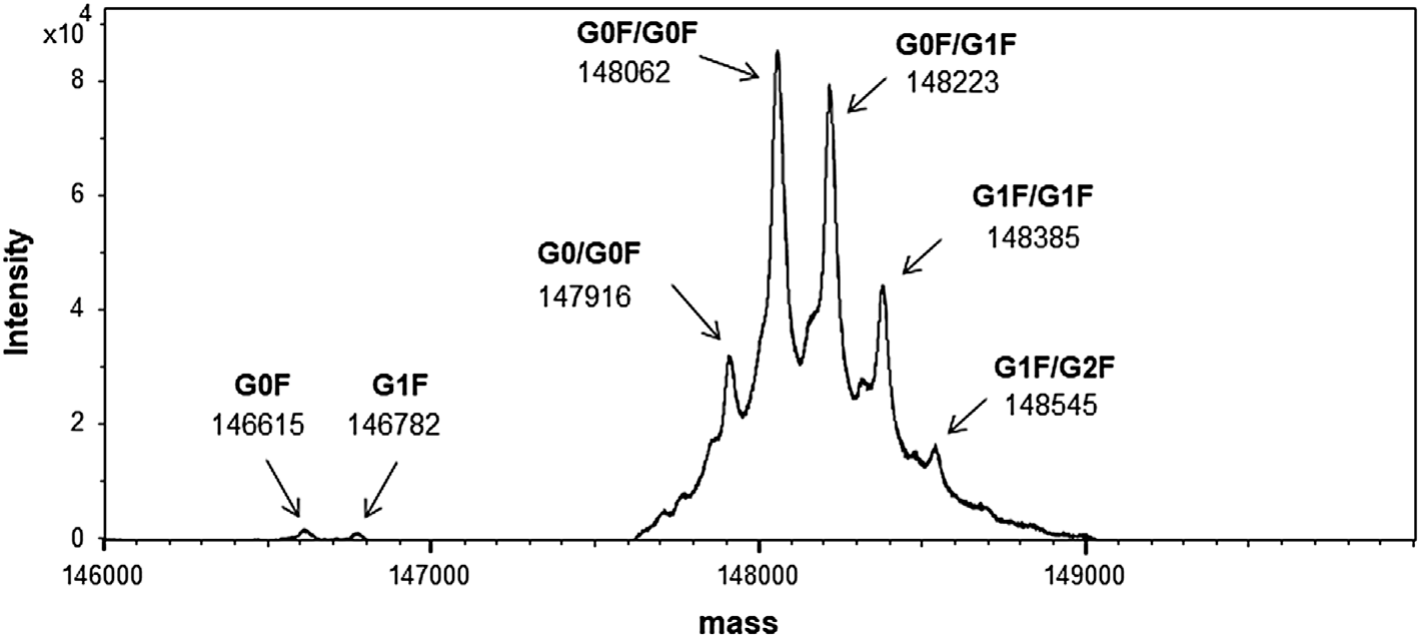
Mass spectrum of the candidate reference material (RM) (intact mass spectrometry). Peaks are shown as averaged mass, and they are assigned based on the glycoform. The calculated and observed masses are summarized in Supplementary Table S1.

Intact mass spectrometry, which can measure the molecular mass of the antibody sample without any sample treatment reveals the molecular mass distribution of the entire molecule along with the major modifications (Lyubarskaya et al., 2006; Brady et al., 2007). The deconvoluted mass spectrum of an intact molecule measured through LC-MS is shown in Figure 4 with the most abundant peak at 148,062 in molecular mass.

Owing to the broad distribution of stable isotopes demonstrated in the IUPAC atomic weight table, the molecular weight was calculated using the average of the minimum and maximum atomic weight values (Wieser and Coplen, 2011). The molecular weight of this material calculated using the atomic weights of 12.0106 (C), 1.00798 (H), 15.9994 (O), 14.0069 (N), and 32.0675 (S) was 148,056, and the molecular formula was C_6560_H_10132_N_1728_O_2090_S_44_, considering a heterotetrameric structure with 16 disulfide linkages and two G0F glycan molecules. Other peaks were also assigned according to the difference in the glycosylation pattern with the calculated mass as summarized in Supplementary Table S1. It was confirmed that one glycan was modified to the antibody molecule in partial glycosylation.

#### 3.2.2 Structure of Light Chain and Heavy Chain

The two main subunits, LC and HC were obtained by reduction of IgG with TCEP, and were analyzed *via* LC-MS. The deconvoluted mass spectrum of HC is shown in Supplementary Figure S1 with the assignment of the glycan structure. The calculated masses and the observed masses of LC and HC are summarized in Supplementary Table S2.

#### 3.2.3 Middle-Down Structural Characterization

**FIGURE 5:**
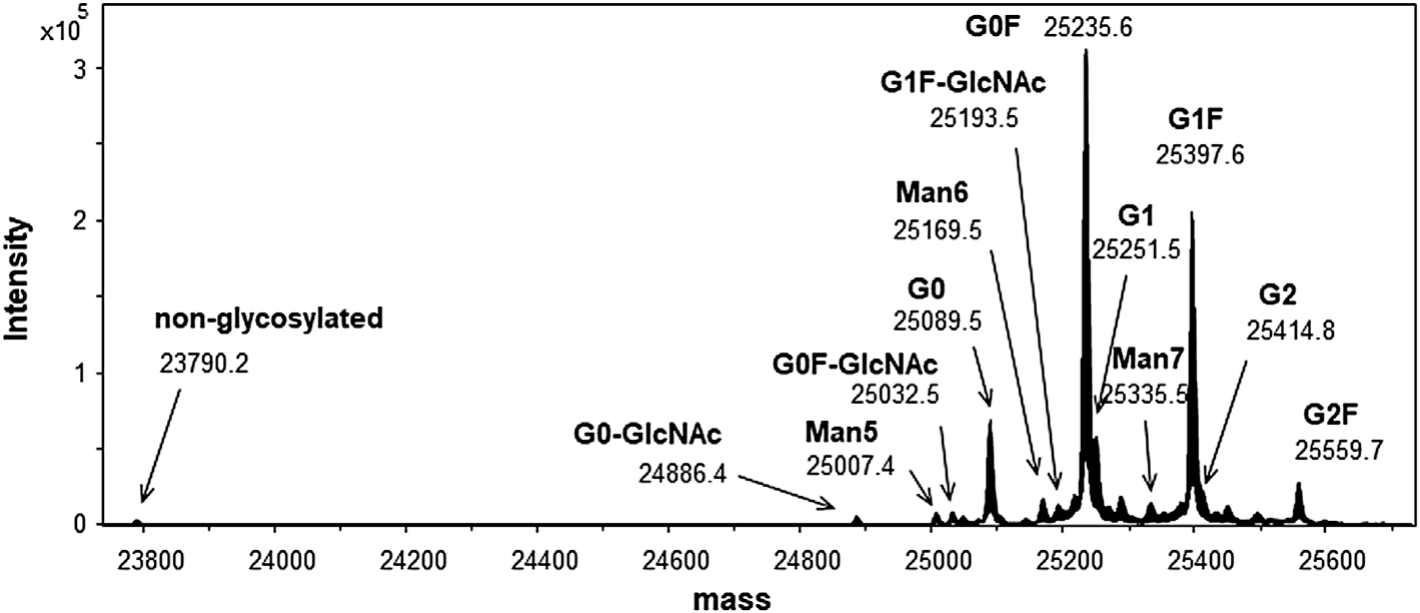
Mass spectrum of scFc fragment obtained by IdeS digestion. Peaks are shown as averaged mass. The calculated and observed masses are summarized in Supplementary Table S3.

For further structural analysis, middle-down structural characterization was performed by investigating the structure of IdeS protease-digested subunits *via* LC-MS (Chevreux et al., 2011; Wang et al., 2013). IdeS protease digests specifically at a single recognition site below the hinge region of the human IgG to provide two molecules, F (ab)’ and scFc. F (ab)’ consists of 1–239 of HC and LC *via* disulfide bond linkage, and scFc is a part of HC with 240–449 (C-terminal end). These IdeS digested products were reduced using DTT to yield Fd’ with 1–239 of HC and LC from F (ab)’, and scFc. The reduced pool was analyzed *via* LC-MS, and the deconvoluted mass spectrum of scFc is shown in Figure 5.

The scFc domain possessed *N*-glycosylation site, and the small size of fragment facilitated the observation of the detailed PTM *e.g.* glycosylation pattern. The calculated and observed masses of scFc as well as F (ab)’ and Fd’ are summarized in Supplementary Table S3. The measurement of the range of molecular masses of scFc, can be performed at a resolution that allows sufficient separation of the isotope peaks in the mass spectrum. Therefore, we identified 12 glycan structures with scFc and the non-glycosylated form and confirmed the structure of scFc.

### 3.3 Glycan Mapping

**FIGURE 6:**
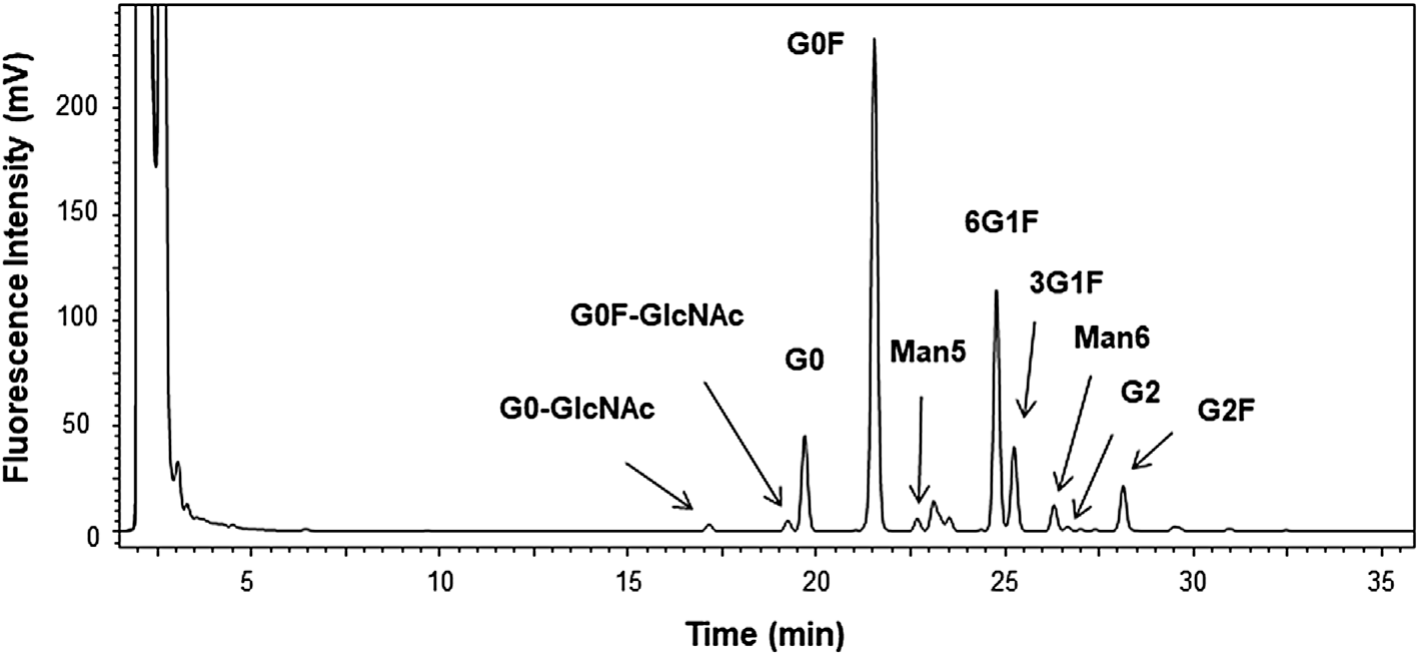
Chromatogram of 2-aminobenzamide–derivatized glycan by liquid chromatography–fluorescent detection (LC-FL) performed as glycan mapping. Glycan was released using peptide *N*-glycosidase F from the candidate reference material (RM). Peaks are assigned for 10 of the peaks with greater than 0.1% in the relative peak area ratio. The relative area ratios are summarized in Supplementary Table S4.

*N*-linked glycans of the candidate RM were analyzed by glycan mapping using LC-FL for the 2-AB–derivatized glycans. Various sample preparations including glycan release with PNGaseF, derivatization with 2-AB and clean-up were conducted using a commercial preparation kit. The chromatogram obtained *via* LC-FL is shown in Figure 6.

The area percentages of triplicate measurements are summarized in Supplementary Table S4. The major assigned 10 peaks were from typical hybrid and high mannose types of *N*-linked glycan of monoclonal antibody produced by CHO cells (Hossler et al., 2009). Based on the results of glycan mapping and analysis of scFc in the previous section, we identified 12 major glycoforms. However, 20 peaks with the relative area ratios greater than 0.1% were observed on the chromatogram, and almost half of the structures are currently unknown. Reliable absolute quantification of glycans has been difficult, and it is necessary to conduct more in-depth analysis in the future by accumulating data through inter-method comparisons and collaborative measurements for structural analysis of unidentified glycans and precise quantification (Wada et al., 2007; De Leoz et al., 2020). The use of recently available certified reference material for glycan analysis, NIST SRM 3655, glycans in solution (Lowenthal and Phinney, 2021) enables highly reliable and accurate analysis.

### 3.4 Peptide Mapping

**FIGURE 7:**
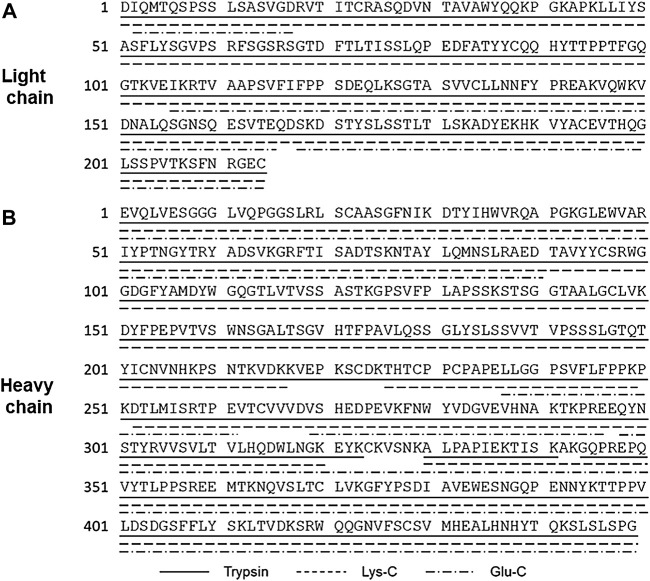
Summary of peptide mapping of light chain **(A)** and heavy chain **(B)** using trypsin, Lys-C, and Glu-C. Peptide identification is summarized in Supplementary Table S5.

Peptide mapping is a widely used bottom-up technique for identifying conformation of the primary structure and modifications such as PTM, N/C-terminal extension, and truncation (Beck et al., 2013). Peptide mapping typically involves denaturation, reduction, and alkylation of protein prior to enzymatic digestion, followed by separation and analysis of the resulting peptide mixture by reversed phase LC-MS/MS (Ren et al., 2009; Lauber et al., 2013; Mouchahoir and Schiel, 2018). For peptide mapping of the candidate RM, enzymatic digestion was performed using trypsin, Lys-C, and Glu-C with different sequence specificities to achieve the highest possible sequence coverage. The results of peptide mapping using these three proteases under reduced condition are presented in Figure 7A for LC and (B) for HC, and the results with the retention times, calculated masses (monoisotopic masses), and observed masses for the identified peptides are summarized in Supplementary Table S5A–C.

Trypsin is the most commonly used protease because of the strict substrate specificity, high activity, and the favorable size of peptides yielded for mass spectrometry (Olsen et al., 2004). Digested peptide fragments generated using trypsin covered most of the entire sequence, but small peptides consisting of a few residues were undetectable. However, peptide mapping with Lys-C and Glu-C digestion in addition to trypsin digestion complementarily and successfully covered the entire sequence. The peptide mapping with the three proteases confirmed the amino acid sequence of the candidate RM to be exactly as designed.

Furthermore, peptide mapping by trypsin digestion revealed the modified structure including N and C terminal structure of HC, deamidation on asparagine residues, oxidation on methionine residues, as well as glycosylation site. The results of peptide mapping regarding these modifications are summarized in Table 1.

**TABLE 1:** Summary of peptide mapping for modified structure including N and C terminal, deamidation, and oxidation *via* trypsin digestion. Calculated and observed masses represent monoisotopic masses.

Structure	Retention time, min	Calculated mass	Observed mass, *m/z*	Charges
Pyroglutamylation at N-terminal	48.6	932.5003	932.4995	2
H1-19, heavy chain				
Lys addition at C-terminal	13.5	394.7298	394.7289	2
H443-449, heavy chain				
Deamidation on N30	23.0	664.6661	664.6650	3
L25-42, light chain				
Deamidation on N55	11.2	543.2673	543.2664	2
H51-59, heavy chain				
Deamidation on N84	25.4	656.3223	656.3214	2
H77-87, heavy chain				
Deamidation on N318	60.7	603.6689	603.6686	3
H305-320, heavy chain				
Deamidation on N387 or 392 or 393	19.5	660.3568	660.3557	3
H374-395, heavy chain				
Oxidation on M107	54.6	934.0907	934.0899	3
H99-124, heavy chain				
Oxidation on M255	48.1	426.2188	426.2179	2
H252-258, heavy chain				

Peptide mapping by trypsin digestion showed partial N-terminal pyroglutamylation of the HC and addition of a lysine residue at the C-terminus of the HC. For deamidation of asparagine residues, the isotopic patterns of Asn and deamidated Asn overlapped when high-resolution mass spectrometry was used. Therefore, peptides with variation in both mass and retention time were determined to be deamidated. The results showed that N30 at the LC, and N55, N84, N318, N387, N392, or N393 at the HC were deamidated. A tryptic peptide, H374-395 carried three asparagine residues (N387, N392, and N393) in the peptide chain. Although we were unable to identify the deamidation site from these data, N387 was a part of the -Asn-Gly- sequence, which is known to be susceptible to deamidation, and those deamidation has been reported (Zhang et al., 2014; Huang et al., 2016). Therefore, N387 could be deamidated among the three asparagine residues. Oxidized methionine residues were found at M107 and M255 of HC.

It is known that IgG1 forms 16 pairs of disulfide bonds to form a heterotetrameric structure with two light chains and two heavy chains (Liu and May 2012). For the analyses of disulfide bond formation, trypsin digestion was performed under non-reducing condition by skipping the reduction and alkylation steps, and the results are summarized in Table 2.

**TABLE 2:** Summary of peptide mapping for structural analysis of disulfide bond formation **(A)**. Identified peptides with a disulfide bond **(B)** amino acid sequence of peptide shown in **(A)**. Tryptic digestion was performed under non-reducing conditions. Calculated and observed masses are shown as monoisotopic mass. L and H represent light chain and heavy chain, respectively.

**(A)** Identified crosslinking peptides *via* disulfide bond					
Peptide	Disulfide linkage	Retention time, min	Calculated mass	Observed mass, *m/z*	Charges *z*
L19-24/L67-103	C23-C88	57.6	964.8563	964.8556	5
L127-142/L191-267	C134-C194	48.9	712.1576	712.1572	5
H20-30/H88-98	C22-C96	26.9	597.0272	597.0266	4
H137-150/H151-213	C147-203	68.3	1320.4944	1320.4938	6
H259-277/H324-325	C264-324	27.2	583.0323	583.0314	4
H364-373/H420-442	C370-428	37.6	769.9725	769.9721	5
L208-214/H222-225	C214(L)-C223(H)	4.7	421.1699	421.1692	3
H226-251/H226-251	C229(H1)-C229(H2)	65.4	910.1384	910.1380	6
	C232(H1)-C232(H2)				

**(B)** Amino acid sequence of peptide forming disulfide linkage shown in **(A)**	
L19-24	VTITCR
L67-103	SGTDFTLTISSLQPEDFATYYCQQHYTTPPTFGQGTK
L127-142	SGTASVVCLLNNFYPR
L191-267	VYACEVTHQGLSSPVTK
L208-214	SFNRGEC
H20-30	LSCAASGFNIK
H88-98	AEDTAVYYCSR
H137-150	STSGGTAALGCLVK
H151-213	DYFPEPVTVSWNSGALTSGVHTFPAVLQSSGLYSLSSVVTVP-SSSLGTQTYICNVNHKPSNTK
H226-251	THTCPPCPAPELLGGPSVFLFPPKPK
H259-277	TPEVTCVVVDVSHEDPEVK
H324-325	CK
H364-373	NQVSLTCLVK
H420-442	WQQGNVFSCSVMHEALHNHYTQK

**FIGURE 8:**
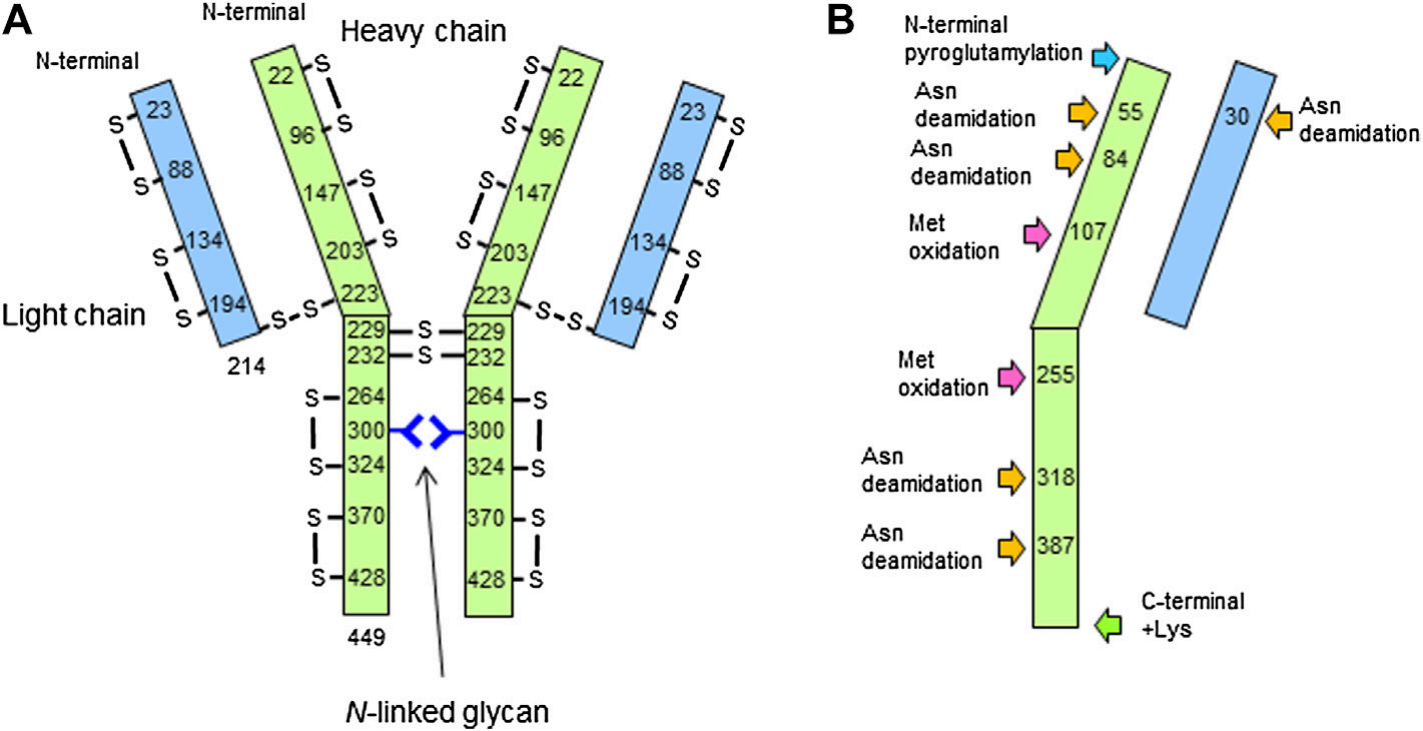
Structure of the candidate reference material (RM) determined by this study. Size of light chain (LC) and heavy chain (LC), disulfide bond formation, and glycosylation site **(A)**, as well as N/C-terminal modifications, Asn deamidation, and Met oxidation **(B)** are described.

The peptide mapping under non-reducing condition successfully revealed disulfide bond sites within the LC and HC, between the LC and HC, as well as between the heavy chains. Based on the above results, the structure determined by mass spectrometry–based analyses is shown in Figure 8.

### 3.5 Homogeneity and Stability of the Candidate RM

Assigning the indicative value (antibody concentration) of candidate RM was performed according to the NMIJ quality system in accordance with ISO 17025 and 17034. Assessing the homogeneity and stability is essential to ensure consistency of the indicative value, and it is mandatory to state their conformity to the international standards.

Homogeneity was assessed through three repeat measurements of the relative area percentage of the main peak obtained by CEX. 12 vials taken by stratified random sampling from whole batch were used for this test. The homogeneity data obtained by analysis of variance (ANOVA) showed that the mean square between-vial variance could not be obtained because of larger value of the variance of within-vial. Therefore, the uncertainty associated with the inhomogeneity was estimated using Eq. 1, considering the measurement variability described in ISO Guide 35 (ISO Guide 35, 2017).

$$u_{bb}=\sqrt{\frac{MS_{within}}{n}}\times \sqrt[{4}]{\frac{2}{\nu MS_{within}}}$$ (1)

where *MS*_within_ represents the mean square within-vial variance, *n* represents number of measurement replicates per vial, *ν* represents degree of freedom of *MS*_within_.

The calculated *u*_bb_ according to Eq. 1 was 0.103%, and this value was used as the relative standard uncertainty.

The stability was evaluated as acceleration, long-term, short-term stabilities, and free-thaw cycle tests *via* CEX. The acceleration test was performed at 4 and 25°C by CEX, SEC, and UV absorption at 280 nm. Time course of the relative area percentage of the main peak obtained by CEX is shown in Supplementary Figure S2A at 4°C and (B) at 25°C. The area ratio of the main peak obtained by CEX decreased after 10 days at 4°C and 3 days at 25°C. Monomer peak ratio on SEC analysis decreased only by 0.8% in 40 days at 25°C (data not shown). No change was observed by UV absorption for 122 days at 4°C and for 40 days at 25°C (data not shown). These results indicated that CEX was the most sensitive method for monitoring the stability.

**FIGURE 9:**
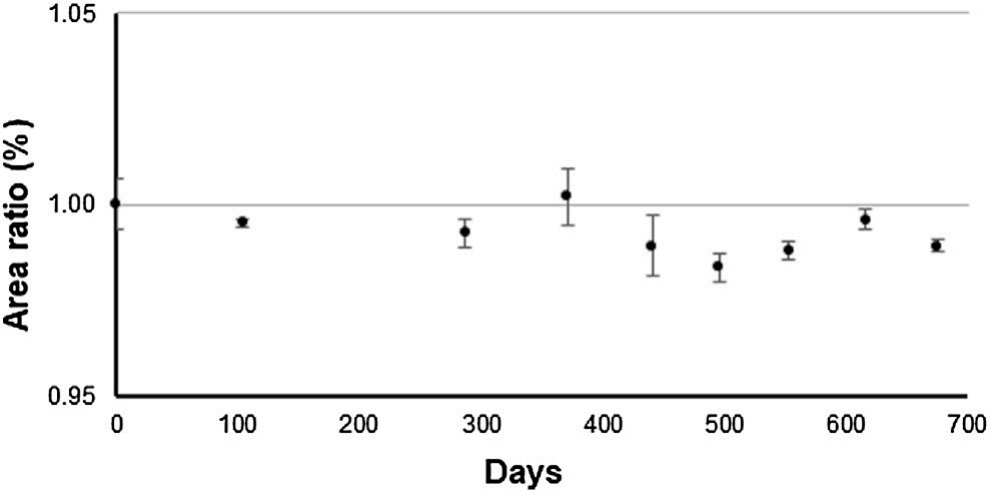
Time course of the main peak of the antibody solution of previous production batch stored at −80°C by triplicate measurement using cation exchange chromatography. *Y*-axis represents the relative peak area ratio of the main peak. This measurement was performed as a long-term stability test.

The long-term stability test was performed using an antibody solution from the previous production batch produced using same production process, and it was monitored for up to 675 days at −80°C *via* CEX. The area ratio of the main peak by CEX over the monitoring period is shown in Figure 9.

A trend analysis was performed according to ISO Guide 35 to determine whether a linear approximation to instability would show a trend in the data over the monitoring period. The result indicated that the significance of the linear regression was not found. By multiplying the uncertainty derived from the slope of the linear regression by 3 years, the uncertainty associated with long-term stability was determined to be 0.923%. The short-term stability was monitored for up to 304 days at −20°C *via* CEX (data not shown). The results of the trend analysis over the monitoring period according to ISO Guide 35 indicated that the significance of the linear regression was not found. The uncertainty associated with the short-term stability was determined to be 0.955% based on the uncertainty derived from the slope of the linear regression.

In addition, freeze-thaw cycle tests were examined as follows: The candidate RM stored at −80°C was moved from a freezer and placed in an incubator at 25°C for 1 h and then returned to the freezer at −80°C. The freeze-thaw cycle test was conducted by repeating this cycle up to five times using CEX, SEC, and UV absorption at 280 nm. The results of the test showed no significant difference over five-times as summarized in Supplementary Table S6, and it can be concluded that freeze-thaw up to five times does not affect the results of any of the methods examined, as well as the indicative value.

### 3.6 Value Assignment of Indicative Value *via* Amino Acid Analyses

Antibody concentration, which is as an indicative value of the RM, was determined *via* amino acid analyses. To ensure the reliability of the amino acid analysis, hydrolyses with hydrochloric acid by microwave-assisted liquid phase (liquid phase) and gas phase were performed independently, and isotope dilution mass spectrometry was used to determine the hydrolyzed amino acids. Amino acid analysis requires complete hydrolysis of the sample into its constituent amino acids. Because the optimal conditions for hydrolysis vary depending on the sample protein, we examined the optimal conditions for the candidate RM (Burkitt et al., 2008; Feng et al., 2020). The hydrolysis conditions were examined at 150, 160, and 170°C for 1 and 3 h for the liquid phase hydrolysis and 130 and 150°C for 18, 24, and 48 h for the gas phase hydrolysis. The optimum conditions were 160°C for 3 h for microwave-assisted liquid phase hydrolysis and 150°C for 48 h for gas phase hydrolysis based on the highest recovery of the eight measured amino acids and the smallest difference between the measured amino acids.

Hydrolyses of the candidate RM were performed under the optimized condition on three separate days and three repeated measurements using LC-MS/MS. The measured concentrations for each amino acid were divided by the number of the amino acid residues in the candidate RM. The quantitative results of each amino acid along with the associate uncertainties are summarized in Table 3 for the liquid phase hydrolysis and for the gas phase hydrolysis. The detailed uncertainty budget is shown in Supplementary Table S7.

**TABLE 3:** Quantitative results of the candidate reference material (RM) by amino acid analyses obtained from liquid phase hydrolysis **(A)** and gas phase hydrolysis **(B)**. The detailed uncertainty budgets are summarized in Supplementary Table S7.

**(A)** Results obtained from liquid phase hydrolysis–amino acid analysis (nmol/g)								
Measured amino acid	Asp	Glu	Pro	Val	Ile	Leu	Phe	Ala
Measured concentration	33.39	33.89	33.42	33.48	34.18	33.58	33.90	34.22
Standard uncertainty	0.1889	0.3454	0.3256	0.2482	0.3628	0.2207	0.5399	0.4881

**(B)** Results obtained from gas phase hydrolysis–amino acid analysis (nmol/g)								
Measured amino acid	Asp	Glu	Pro	Val	Ile	Leu	Phe	Ala
Measured concentration	32.92	33.94	33.76	33.77	33.96	33.71	33.75	35.97
Standard uncertainty	0.7506	0.2116	0.1611	0.1452	0.2349	0.3823	0.2684	0.4297

The protein concentration was obtained by calculating the weighted mean of the quantitative results of the eight amino acids. From the calculation, the concentrations of the candidate RM obtained *via* liquid phase and gas phase hydrolyses were (33.68 ± 0.42) nmol/g and (33.94 ± 0.98) nmol/g, respectively (the number following ± represents the standard uncertainty.).

The validity of the analyses was confirmed using certified reference material of human serum albumin (NMIJ CRM 6202a) with a certified value of the concentration (74.3 ± 3.2) g/L (expanded uncertainty with coverage factor, *k* = 2) (Kinumi et al., 2017). The quantitative results by liquid phase and gas phase hydrolyses were (74.4 ± 0.9) g/L and (74.1 ± 0.8) g/L, respectively (the number following ± represents the standard deviation.). This indicates that the amino acid analysis was sufficiently accurate and suitable for the value assignment.

### 3.7 Impurity Assessment and its Influence on the Result of Amino Acid Analyses

In addition, we assessed protein, peptide, and amino acid impurities in the candidate RM which may affect the result of the amino acid analyses. The raw material was highly purified by three-step-chromatography as equivalent to that used in the production of biopharmaceutical. Possible impurities should be considered are HCP and protein A, which could be contaminated during the purification process (Hogwood et al., 2014). The quantitative results of HCP and protein A by ELISA were 3.2 ng/mg protein and 0.2 ng/mg protein respectively, which were significantly low, and did not affect the results of amino acid analyses of the material. To assess peptide and free amino acid contaminants, amino acid analysis was performed for hydrolysate by hydrochloric acid hydrolysis of filtrates with a 10 kDa ultrafiltration device (Amicon ultra, Millipore, USA) for the candidate RM and phosphate buffer (as the blank sample). The quantitative results indicated that contents of the eight measured amino acid in the filtrate of the candidate RM were equivalent or lower compared to those of the blank. These results indicate no impurities that could affect the results of amino acid analysis, and the values obtained can be corresponded to the concentration of antibody.

Furthermore, quantitative analysis was performed for host cell–derived DNA and endotoxin which are often considered process-related impurities, and their quantification results were below the lower limit of quantification (Host cell–derived DNA, 10.0 pg/mg protein; endotoxin, 0.01 EU/mg protein).

### 3.8 Establishing the Indicative Value

As the measurand is a heterotetrameric structure including oligomeric forms, the indicative value can be determined by using the results of amino acid analysis. The concentration of the candidate RM was calculated from the weighted mean of the results of the amino acid analyses by liquid phase and gas phase hydrolyses, which was to be 33.75 nmol/g. Finally, the indicative value of the candidate RM was determined to be 5.00 g/L by converting the unit to mass concentration using the molecular weight (148,056) and the density (1.0008 g/cm^3^ at 20°C) measured by a vibration-type density meter. The molecular weight was calculated based on the amino acid sequence with 16 disulfide linkages and the most abundant glycan structure (G0F/G0F) described in previous section.

The uncertainty associated with the concentration from two amino acid analyses was calculated according Eq. 2 (Kinumi et al., 2017):

$$u^{2}\left(x\right)=\sum w_{i}^{2}u^{2}\left(x_{i}\right)+Dif^{2},$$ (2)

where *w*_i_ represents the weight of the quantitative results by liquid phase and gas phase hydrolyses, *u* (x_i_) represents the uncertainty of the quantitative results by the two hydrolysis methods, and *Dif* represents the difference between the two quantitative methods calculated as a rectangular distribution of the two quantitative results. From the equation, the standard uncertainty associated with amino acid analysis was calculated to be 0.19 g/L.

In conclusion, the uncertainty budget is summarized in Table 4 according to ISO Guide 35, considering the following uncertainty components: amino acid analysis for value assignment, inhomogeneity of the material, long-term instability, and short-term instability. The uncertainty estimation of the indicative value of the candidate RM was calculated as the combined standard uncertainty by summing the squares of the uncertainty components listed in Table 4.

**TABLE 4:** Uncertainty budget for the indicative value of NMIJ RM 6208a, AIST-MAB.

Uncertainty components (%)	
Amino acid analysis	1.30
Inhomogeneity	0.10
Instability (long-term)	0.92
Instability (short-term)	0.95
Combined standard uncertainty (Rel, %)	1.86
Expanded uncertainty (*k* = 2) (g/L)	0.19

Overall, the indicative value of the candidate RM was determined to be (5.00 ± 0.19) g/L, and the number following ± represents the expanded uncertainty (coverage factor *k* = 2).

### 3.9 Extinction Coefficient at 280 nm

Because the absorbance of the protein solution can be measured using a low cost instrument with high reproducibility and simple measurement principle based on the Lambert-beer’s law, the absorbance at 280 nm is frequently used to determine antibody concentration (Miranda-Hernandez et al., 2016; Cole et al., 2018). Antibody concentration is essential in the quality control of an antibody drug, and the accurate determination of the extinction coefficient at 280 nm of the RM can lead to various application of the RM with high accuracy.

The extinction coefficient of the candidate RM was obtained according to the Lambert-Beer equation: the absorbance measured by a spectrophotometer at 280 nm using an optical cell with an assessed optical path length; the antibody concentration determined as the indicative value by amino acid analysis. The absorbance of the candidate RM was measured using a quartz cell with an optical path length of 1 mm. Because the absorbance at 280 nm is approximately 0.7 when measured with an optical path length of 1 mm, which is appropriate for accurate measurement. Because the only available optical cell whose optical path length was evaluated was a 10 mm cell supplied by Hitachi Ltd determined to be (10.00 ± 0.05) mm, the extinction coefficient at 280 nm of potassium hydrogen phthalate solution was determined using this 10 mm cell, and the optical path length of the 1 mm cell was obtained using the determined extinction coefficient of the potassium hydrogen phthalate solution. Finally, the optical path length of the 1 mm optical cell was determined to be (1.02 ± 0.01) mm (the number following ± represents the standard uncertainty.). To measure the absorbance of protein solutions containing aggregates, it is necessary to consider the effect of light scattering. The correction of the light scattering at 280 nm was made using the results of absorbance measurements at 320–350 nm according to literature (Maity et al., 2009). Using the indicative value (measured antibody concentration), optical path length, and absorbance at 280 nm using the 1 mm optical cell, the extinction coefficient was found to be (1.41 ± 0.03) L/(g cm) with the correction of light scattering (the number following ± represents the standard uncertainty.). The extinction coefficient of proteins at 280 nm is often predicted using the equation by Pace et al., based on the numbers of tryptophan, tyrosine, and cystine residues in the protein molecule (Pace et al., 1995). The calculated absorption coefficient of the candidate RM is 1.42 L/(g cm), which is consistent with the experimental result. We performed an inter-laboratory comparison on UV measurement at 280 nm for monoclonal antibody solution, and we found that the standard deviation of the measurement results of 34 laboratories was approximately 10%, indicating a large variation among laboratories (Kinumi et al., 2021). It has been shown that this difference in absorbance can be reduced by conducting system suitability test. The use of this material enables validation of absorbance measurement and it can realize more accurate measurement.

### 3.10 Other Physicochemical Properties, Analyses of Particle Size and Higher-Order Structure

The physicochemical properties of the candidate RM regarding its particle size and higher-order structure were also analyzed by SEC-MALS (Supplementary Figure S3), DLS (Supplementary Figure S4), NTA (Supplementary Figure S5), FI (Supplementary Figure S6), CD (Supplementary Figure S7), and TSA (Supplementary Figure S8). The molecular weight calculated from SEC-MALS was 1.35 × 10^5^, indicating that the main peak corresponded to the IgG monomer. The relative UV peak area (94.6%) demonstrated that most of the candidate RM was dispersed as monomeric form. The DLS measurements presented a typical value (11.4 nm) of hydrodynamic diameter for the IgG monomer and no significant peaks for aggregates, which was consistent with the SEC-MALS results. The presence of detectable amounts of submicrometer-sized particles was clarified *via* NTA analysis. The mode diameter, mean diameter, and particle concentration were 194 nm, 206 nm, and 5.81 × 10^8^ particles/mL, respectively. The FI measurements demonstrated that the candidate RM contained considerable number of micrometer-sized particles (e.g., ∼7,000 particles/mL for a particle of ≥5 nm diameter) compared with trastuzumab-US (Hutterer et al., 2019). The CD spectrum of the candidate RM exhibited a typical shape of a globular protein rich in β-sheet structures. The thermal denaturation temperatures revealed by TSA analysis were 69.7 and 80.4°C, which were comparable to the values reported for trastuzumab-US (Hutterer et al., 2019). These results indicate that the candidate RM maintains the higher-order structure of the native IgG.

### 3.11 AIST-MAB and NISTmAb, the Difference and Complementary Information of the Properties

NISTmAb (RM 8671) from NIST is the humanized IgG1κ solution produced using mouse-derived NS0 cells, and assigned the antibody concentration, size heterogeneity, and charge heterogeneity as the reference values. This material also provides with the case study of various properties.

AIST-MAB was produced using CHO cell line, which is used in the production of over 70% of biopharmaceuticals of which almost all of the monoclonal antibodies (Lalonde and Durocher, 2017). The difference in the host cells for the production, can lead to a large difference in properties, especially in the glycan structure. Even both materials are recombinant humanized IgG1κ, the amino acid sequence, buffer formulation, and method used for value assignment in addition to host cells were between them differed. The development of antibody reference material with different properties, specifically produced by the CHO cells, presents considerable advantage in antibody characterization. Conversely, to evaluate the various properties of monoclonal antibodies, orthogonal approaches of analyses using multiple reference materials can provide complementary information and improve the quality of characterization. The reference materials are not exclusive to each other, and the availability of multiple reference materials for antibody characterization could expand the scope of the utilization of each reference material.

## 4 Conclusion

The monoclonal antibody reference material presented here, NMIJ RM 6208a, AIST-MAB provides a tool that enables a wide range of analytical techniques to be extensively compared and evaluated, going beyond quality control with in-house reference materials. The indicative value represents the antibody concentration with associated uncertainty; however, the most of properties and the corresponding results are method-defined. The measurement results of various physicochemical properties with underpinning by accurate antibody concentration are more reliable and allow for promising measurement. Reference materials that can be used across analytical methods and analytical laboratories enable the development and standardization of analytical methods as well as the verification of in-house consistency in measurements. From a metrological point of view, the capability of inter-laboratory comparisons will open up the possibility of development of traceable measurements for various properties relevant to the antibody analysis. The AIST-MAB is currently available from NMIJ/AIST and contributes to the development of more advanced and reliable analytical techniques for antibody characterization.

## Data Availability

The original contributions presented in the study are included in the article/Supplementary Material, further inquiries can be directed to the corresponding author.

## Abbreviations

CD, circular dichroism; CEX, cation exchange chromatography; CHO, Chinese hamster ovary; cIEF, capillary isoelectric focusing; CRM, certified reference material; DLS, dynamic light scattering; DTT, dithiothreitol; ELISA, enzyme-linked immunosorbent assay; FI, flow imaging; GAPDH, glyceraldehyde-3-phosphate dehydrogenase; HC, heavy chain; HCP, host cell–derived protein; ISO, International Organization for Standardization; LC, light chain; LC-FL, liquid chromatography–fluorescent detection; LC-MS, liquid chromatography–mass spectrometry; LC-MS/MS, liquid chromatography–tandem mass spectrometry; NMIJ, National Metrology Institute of Japan; NIST, National Institute of Standards and Technology; NTA, nano tracking analysis; PTM, posttranslational modification; qPCR, quantitative polymerase chain reaction; RM, reference material; SEC, size-exclusion chromatography; SEC-MALS, size exclusion chromatography–multi-angle static light scattering; TCEP, tris(2-carboxyethyl)phosphine; TSA, thermal shift assay; UV, ultraviolet.
