# Supplementary material for: Characterization and Value Assignment of a Monoclonal Antibody Reference Material, NMIJ RM 6208a, AIST-MAB
Source: Front Mol Biosci. 2022 Jun 6;9:842041. doi: 10.3389/fmolb.2022.842041 (PMC9207415; doi:10.3389/fmolb.2022.842041)
Supplement: Supplementary file 1 [file DataSheet1.docx]

*Supplementary Material for*

**Characterization and Value Assignment of a Monoclonal Antibody Reference Material, NMIJ RM 6208a, AIST-MAB**

Tomoya Kinumi^1,2^*****, Kazumi Saikusa^1^, Megumi Kato^1^, Reiko Kojima^2^, Chieko Igarashi^2^, Naohiro Noda^2,3^, and Shinya Honda^2,3^

^1^National Metrology Institute of Japan (NMIJ), National Institute of Advanced Industrial Science and Technology (AIST), Tsukuba, Japan

^2^Manufacturing Technology Association of Biologics (MAB), Kobe, Japan

^3^Biomedical Research Institute, National Institute of Advanced Industrial Science and Technology (AIST), Tsukuba, Japan

**Supplementary Data**

**Measurement condition for amino acid analyses**

**Liquid phase hydrolysis, pre-column derivatization LC-MS/MS measurement:**

The hydrolysate was modified using *N*-butylnicotinic acid *N*-hydroxysuccinimide ester iodide and injected into an LC–MS/MS system with a C30 reversed-phase column (Develosil C30-UG-5, 5 μm, 2 mm diameter × 250 mm length, Nomura Chemical, Japan) and a TSQ Quantum triple quadrupole mass spectrometer (Thermofisher Scientific, USA) with an electrospray ion source. Solvent and gradient conditions: mobile phase A (0.05% trifluoroacetic acid and 0.5% formic acid/ H_2_O) and mobile phase B (0.05% trifluoroacetic acid and 0.5% formic acid/ acetonitrile) under the gradient condition of 5%–20% B in 10 min then 20%–55% B in 10 min at 0.2 mL/min. The SRM transitions were as follows. Asp: 295.13 > 205.13; Glu: 309.15 > 263.14; Pro: 277.16 > 233.17;; Val: 279.17 > 233.17; Leu and Ile: 293.19 > 247.18; Phe: 327.17 > 281.17; Ala: 251.14 > 205.13. Asp*:300.14 > 208.138; Glu*: 315.16 > 268.15; Pro*: 283.17 > 233.17; Val*: 285.19 > 238.18; Leu* and Ile*: 300.20 > 253.20; Phe*: 337.20 > 290.19; Ala*: 255.15 > 205.13, where“*” indicates an isotopically labeled amino acid.

**Gas phase hydrolysis, HILIC-MS/MS measurement:**

The hydrolysate was injected into an LC–MS/MS system with a ZIC-HILIC column (2.1 mm diameter × 250 mm, Merk Chemicals, Germany) and a 8040 Quantum triple quadrupole mass spectrometer (Shimadzu, Japan) with an electrospray ion source. Solvent and gradient conditions: mobile phase A (10 % acetonitrile, 8 mmol/L ammonium formate, 0.12 % formic acid/ H_2_O) and mobile phase B (90 % acetonitrile, 8 mmol/L ammonium formate, 0.12 % formic acid/ H_2_O) under the gradient condition of 94 %–88 % B in 6 min, 88 %–50 % B in 5 min, then 50 % B in 3 min at 0.4 mL/min. The SRM transitions were as follows. Asp: 134.05 > 73.90; Glu: 148.05 > 84.05; Pro: 116.10 > 70.00; Val: 118.10 > 72.05; Leu and Ile: 132.15 > 86.05; Phe: 166.05 > 120.05; Ala: 90.00 > 44.00. Asp*:139.05 > 76.95; Glu*: 154.05 > 89.05; Pro*: 122.10 > 75.00; Val*: 124.15 > 77.05; Leu* and Ile*: 139.10 > 92.05; Phe*: 170.10 > 129.10; Ala*: 94.10 > 47.00, where“*” indicates an isotopically labeled amino acid.

**Materials and Methods for analyses of particle size and higher-order structure**

**Size exclusion chromatography-multi-angle static light scattering (SEC-MALS)**

To characterize the molecular weight distribution, SEC-MALS measurement was performed using Infinity 1220 HPLC system (Agilent Technologies, USA) equipped with a TSK gel G3000SW_XL_ column (5 μm, 7.8 mm diameter × 300 mm length, TOSOH, Japan), DAWN HELEOS II 8+ (Wyatt Technology, USA) as a multi-angle light scattering detector, and Optilab T-rEX (Wyatt Technology, USA) as a refractive index detector. First, 10 μL of the candidate RM solution (2.5 mg/mL) was injected into the column and eluted at a flow rate of 0.6 mL/min at room temperature, with a buffer containing 50 mmol/L sodium phosphate and 200 mmol/L NaCl (pH 7). The UV absorption signal of the eluate was monitored at 280 nm. In MALS analysis, the refractive index increment (d*n*/d*c*) and UV extinction coefficient at 280 nm were 0.185 and 1.4, respectively.

**Dynamic light scattering (DLS)**

The size distribution of nanoscale particles was analyzed using DynaPro Plate Reader-II (Wyatt Technology, USA). Before DLS measurements, filtrated samples with 0.45 μm filter were equilibrated for 2 min at 25 °C. A signal recording of 10 s was repeated 10 times for each measurement, and the accumulated data were averaged. All measurements were performed at 25 °C in triplicate. The viscosity and reflective index of the sample solutions were 0.890 mPa/s (25 ℃) and 1.333 (20 ℃, 589nm), respectively.

**Nano tracking analysis (NTA)**

The size distribution of subvisible particles was analyzed using NanoSight NS500 (Quantum Design, Japan). Sample solutions and standard solutions for a system compatibility test containing 100 μm latex (Nanosphere Size Standard, Thermo Fisher Scientific, USA) were loaded to the sample chamber using an internal pump and subjected to NTA analysis. Each measurement was performed at 25 °C was repeated for thrice. Camera level and recording time were 12 and 60 s, respectively. Data were evaluated using NanoSight NTA2.3 software (Quantum Design, Japan).

**Flow imaging (FI)**

The size distribution of micrometer-sized particles was analyzed using an MFI 5200 (ProteinSimple, USA) equipped with a 100 μm/SP3 flow cell (ProteinSimple, USA). Sample solutions were gently inverted five times, and then subjected to FI measurement. First, 0.2 mL of the sample solution was used for priming the flow cell, and then 0.6 mL of the solution was measured at a flow rate of 0.1 mL/min. Particle images were recorded at 226 fps. Each measurement was repeated thrice. Data were evaluated using a MVSS ver. 1.4 software (ProteinSimple, USA).

**Circular dichroism (CD)**

CD spectra were measured using J-1500 spectropolarimeter (JASCO, Japan) to investigate the secondary structure of a protein. Measurements were conducted at 25 °C using a quartz cell with 1 mm path length. Sample proteins were diluted at 0.1 mg/mL using 10 mmol/L potassium phosphate buffer (pH 7.0). The spectrum of the buffer was recorded as a blank. Four spectra were accumulated and averaged.

**Thermal shift assay (TSA)**

Thermal denaturation of a protein, i.e., collapse of the higher-order structure, was analyzed by the TSA method [alternatively known as differential scanning fluorimetry (DSF)] using the StepOnePlus Real-Time PCR System (Applied Biosystems, USA) with Protein Thermal Shift kit (Applied Biosystems, USA). All reactions were set up in final volumes of 20 µl in 96-well plates. Aliquot in each well contained 0–2.0 μL of sample (ca. 5 mg/mL protein), 2.5 μL of 8× Protein Thermal Shift dye (Applied Biosystems, USA), and 15.5–17.8 μL of 10 mmol/L potassium phosphate buffer (pH 7.0), which were mixed and incubated on ice. Thermal melting measurements were conducted at a ramp rate of 1.6 °C/s and a temperature range of 25 to 99 °C. All experiments were repeated five times. Data were analyzed using the Protein Thermal Shift Software (Applied Biosystems, USA).

**Supplementary Tables**

**Table S1** Peak assignment of intact mass spectrum of candidate reference material shown in Figure 4. Averaged mass is shown in this table.

**Table S2** Peak assignment of light chain (LC) and heavy chain (HC) of candidate reference material shown in Figure S1. Monoisotopic mass in measurement results was estimated by the function of SNAP II implemented in Data Analysis 4.3 (Bruker, Germany), the analysis software of the mass spectrometer.

**Table S3** Peak assignment of F(ab)’, Fd’, and scFc obtained by IdeS digestion of candidate reference material shown in Figure 5.

Monoisotopic mass in measurement results was estimated by the function of SNAP II implemented in Data Analysis 4.3 (Bruker, Germany), the analysis software of the mass spectrometer.

**Table S4** Quantification result of glycan mapping by liquid chromatography-fluorescent detection (LC-FL) shown in Figure 6. Relative peak area ratios by triplicate measurement are shown.

**Table S5** Summary of the identified peptide digested by **(A)** trypsin, **(B)** Lys-C, and **(C)** Glu-C shown in Figure 7. All masses are shown as monoisotopic mass. Cysteine residue is carboxymethylated. L and H represent light chain and heavy chain, respectively. “*” represents missed cleavage. ND: not detected

Table S5 (A) Peptide mapping by trypsin digestion

Table S5 (B) Peptide mapping by Lys-C digestion

Table S5 (C) Peptide mapping by Glu-C digestion

**Table S6** Results of freeze-thaw (FT) cycle tests by monitoring cation exchange chromatography (CEX), size-exclusion chromatography (SEC), and ultraviolet (UV) absorption

The data were normalized to 1.000 of those of 0 times. Measurement results by triplicate measurement with the standard deviation (SD) are shown.

**Table S7** Detailed uncertainty budget for amino acid analyses by **(A)** liquid phase hydrolysis and **(B)** gas phase hydrolysis shown in Table 3.

The associated uncertainties considered the following components: *u*(M within), precision within measurements; *u*(W std), weighing of amino acid standard solution during preparation; *u*(W spike std), weighing of isotopically labeled amino acid that was spiked into the amino acid standard solution; *u*(W spike prep), weighing of isotopically labeled amino acid that was spiked into the sample solution; *u*(Purity AA), purity of the amino acid; *u*(Cal curve), calibration curve.

**(A)** Uncertainty budget for amino acid analysis via liquid phase hydrolysis

**(B)** Uncertainty budget for amino acid analysis via gas phase hydrolysis

**Supplementary Figures**

**
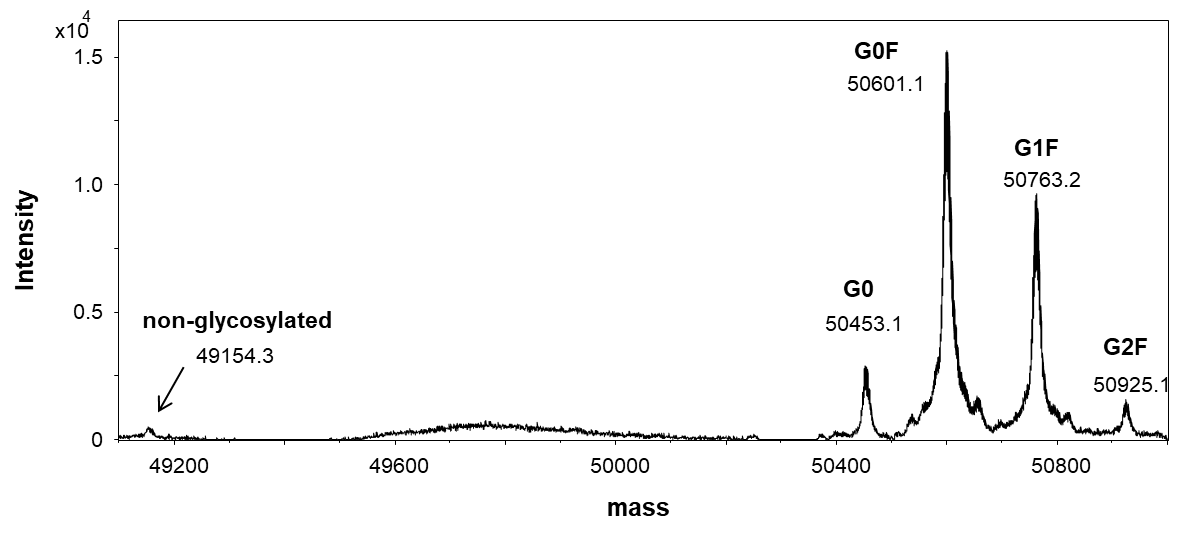
**

**Figure S1.** Mass spectrum of heavy chain of the candidate reference (RM) obtained by reduction. Peaks are shown as averaged mass and they are assigned based on the glycoform. The calculated and observed masses are summarized in Table S2.

**
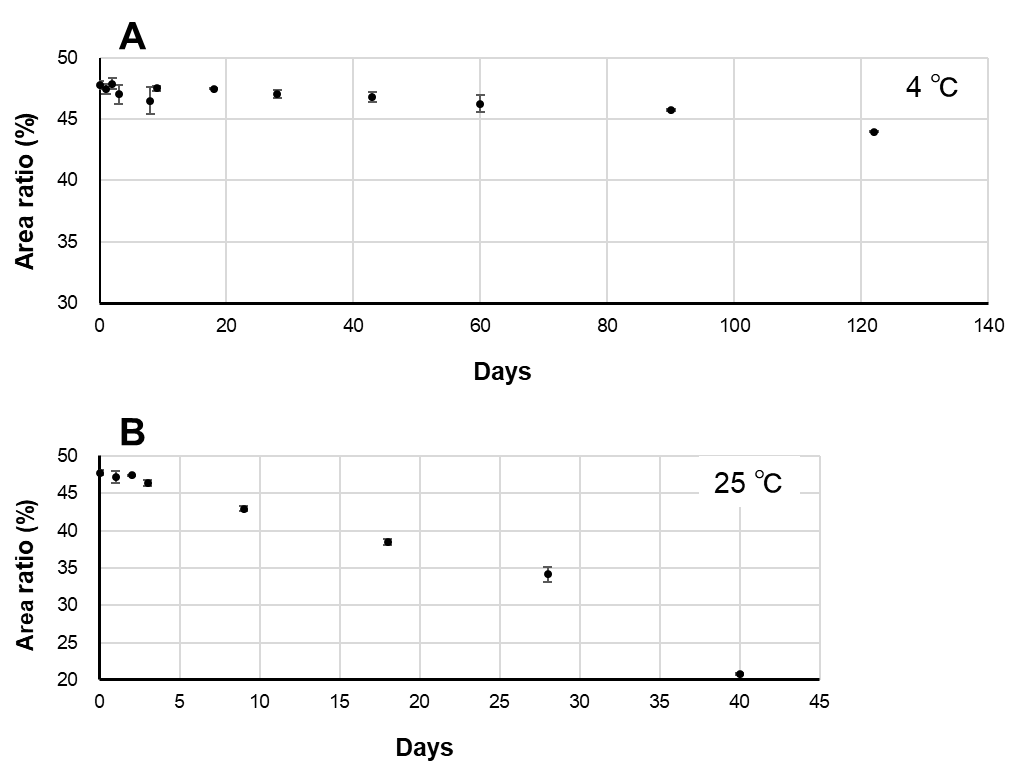
**

**Figure S2**. Time course of the main peak of the candidate reference material (RM) stored at 4 °C **(A)** and 25 °C **(B)** by triplicate measured using cation exchange chromatography. Y-axis represents relative peak area ratio of the main peak. This measurement was performed as an acceleration test.

**
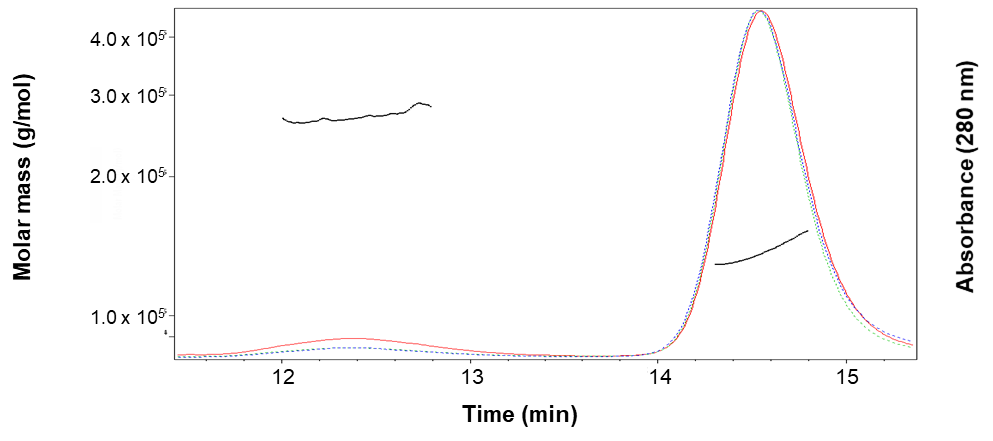
**

**Figure S3**. Molecular weight distribution analyzed by size exclusion chromatography - multi-angle static light scattering (SEC-MALS) analysis.

**
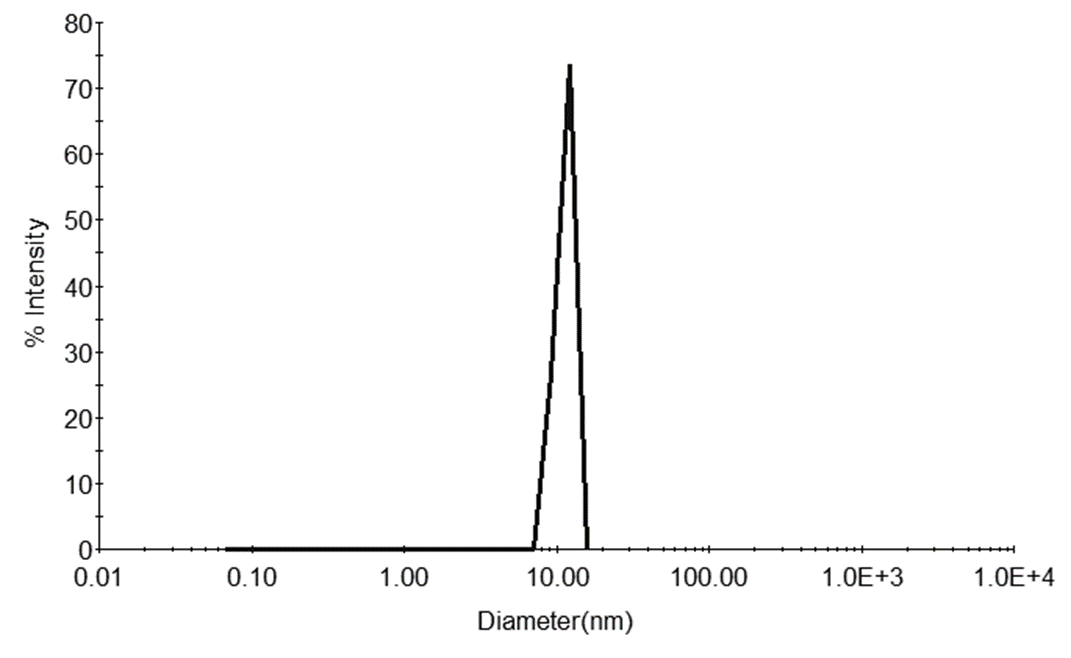
**

**Figure S4**. Size distribution of nanoscale particle analyzed by dynamic light scattering (DLS).


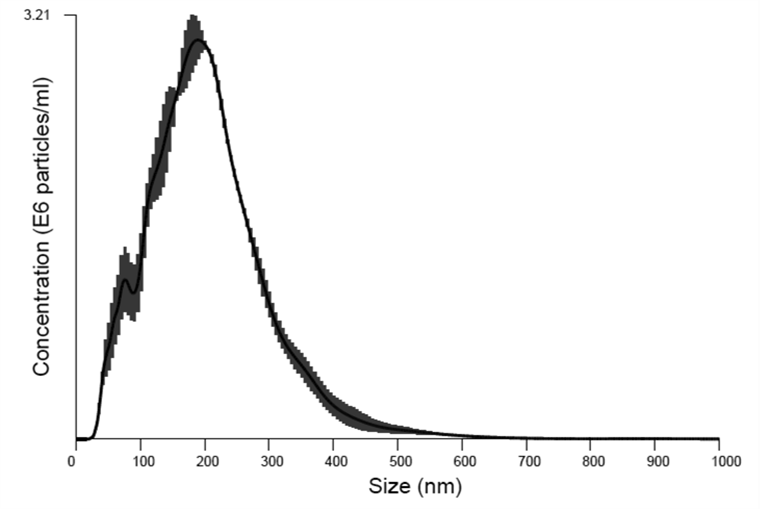


**Figure S5**. Size distribution of subvisible particles analyzed by nano tracking analysis (NTA).

**
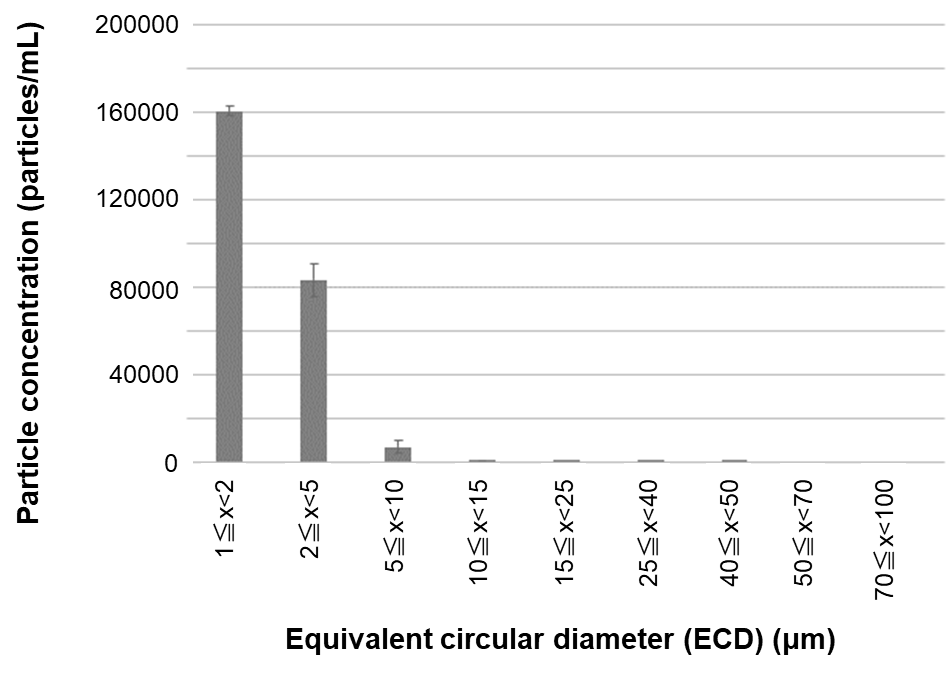
**

**Figure S6**. Size distribution of micrometer-sized particle analyzed by flow imaging (FI).


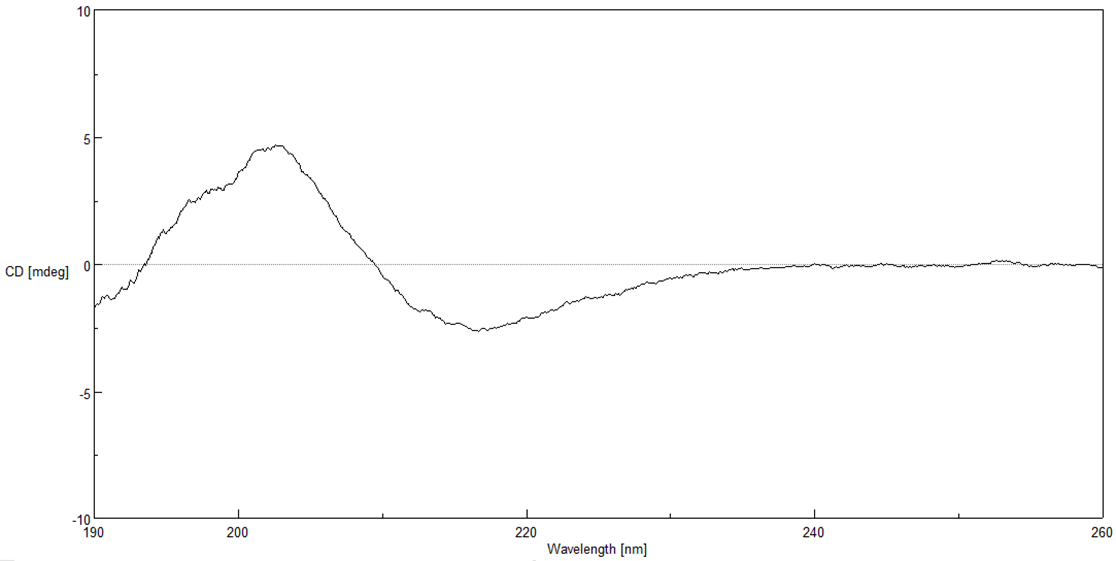


**Figure S7**. Circular dichroism (CD) spectrum of candidate reference material


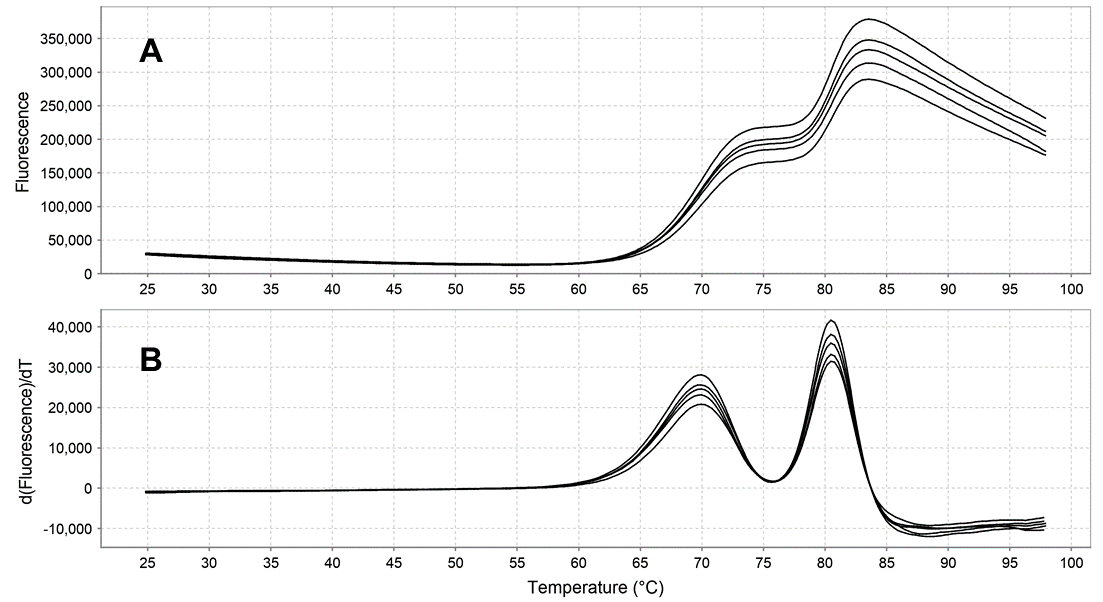


**Figure S8**. Thermal shift assay (TSA) of candidate reference material. (A) and (B) represent the raw data and the differential curve, respectively.
